# Benefits, risks, barriers, and facilitators to cycling: a narrative review

**DOI:** 10.3389/fspor.2023.1168357

**Published:** 2023-09-19

**Authors:** Greig Logan, Camilla Somers, Graham Baker, Hayley Connell, Stuart Gray, Paul Kelly, Emma McIntosh, Paul Welsh, Cindy M. Gray, Jason M. R. Gill

**Affiliations:** ^1^ School of Cardiovascular and Metabolic Health, University of Glasgow, Glasgow, United Kingdom; ^2^ Institute of Health and Wellbeing, University of Glasgow, Glasgow, United Kingdom; ^3^ Physical Activity for Health Research Centre, University of Edinburgh, Edinburgh, United Kingdom

**Keywords:** cycling, physical activity, active travel, public health, economic

## Abstract

There is large potential to increase cycling participation worldwide. Participation in cycling is associated with lower risk of mortality from any cause, and incidence of cardiovascular disease and type 2 diabetes, as well as positive mental health and well-being. The largest potential for health gains likely to come from increasing participation amongst those who do not currently cycle regularly, rather than encouraging those who already cycle regularly to cycle more. Replacing car journeys with cycling can lead to reductions in air pollution emissions and lower pollutant exposure to the general population. Important gaps and uncertainties in the existing evidence base include: the extent to which the health benefits associated with cycling participation are fully causal due to the observational nature of much of the existing evidence base; the real-world economic cost-benefits of pragmatic interventions to increase cycling participation; and the most effective (combination of) approaches to increase cycling participation. To address these uncertainties, large-scale, long-term randomised controlled trials are needed to: evaluate the effectiveness, and cost-effectiveness, of (combinations of) intervention approaches to induce sustained long-term increases in cycling participation in terms of increases in numbers of people cycling regularly and number of cycling journeys undertaken, across a range of population demographic groups; establish the effects of such interventions on relevant outcomes related to health and wellbeing, economic productivity and wider societal impacts; and provide more robust quantification of potential harms of increasing cycling participation, such as collision risks.

## 1. Introduction

Established causal relationships between general physical activity and health outcomes are well documented (1, 2). While riding bicycles is often included in population measures of physical activity alongside other exercise modalities, few studies have investigated the contribution of cycling alone with health associations (3). Cycling can be performed for leisure, sport, commuting, active transport and utility (e.g., shopping, school run), however quantification of precise contributions of cycling purpose to health are not well known (4). Despite this, cycling is increasingly forming an important component of public health recommendations and active transport policy, but uncertainty still exists about the effectiveness of intervention strategies to improve cycling (5).

This narrative review intends to provide a balanced, evidence-based, overview of the benefits and risks of cycling and the potential scope of consequences of increasing cycling participation for health, wellbeing, the environment, and the economy, as well as providing an overview of the evidence about barriers to cycling, and approaches which have been tried so far to increase cycling participation. Throughout, we aim to summarise what is known on each of these topics and to highlight the key evidence gaps and to outline the next steps needed to address these gaps. In doing so, we provide a clear pathway, outlining the work still needed, to facilitate the goal of a sustained increase in cycling uptake globally.

## 2. The benefits of cycling on physical health outcomes

For outcomes where relevant systematic reviews and meta-analyses have been published, findings from these analyses have been reported. These have been updated with of evidence from more recent studies published since these reviews reported as appropriate. In areas where a systematic review or meta-analysis has not been published, we have attempted to report a representative overview of the available data. The available evidence on cycling and specific health outcomes is summarised in the sections below.

### 2.1. Cycling and risk of mortality

The available evidence from large prospective cohort studies indicates that regular cycling is associated with a lower risk of mortality. In 2014, Kelly et al. published a comprehensive systematic review and meta-analysis collating evidence from all published studies in healthy adults investigating the association between participation in cycling and risk of mortality (3). This analysis included data from seven large scale studies—four from Denmark, two from the United Kingdom, and one from China—which included approximately 200,000 adults aged from 20 to 93 years, and over 2 million person-years of observation. In these studies, participation in cycling was assessed and participants were followed up for between 5.7 and 18 years with mortality outcomes over this follow-up period recorded. The data were statistically adjusted for a range of potential confounding factors. All studies performed adjustment for age, smoking, other non-cycling physical activity, at least one indicator of socio-economic status, and aspects of health status, and either adjusted for sex or performed analyses in single sex groups. Four studies also adjusted for body mass index, and three studies adjusted for alcohol intake. Key features of the studies included in the 2014 Kelly meta-analysis (3) are outlined in Supplementary Table S1.

**Figure 1:**
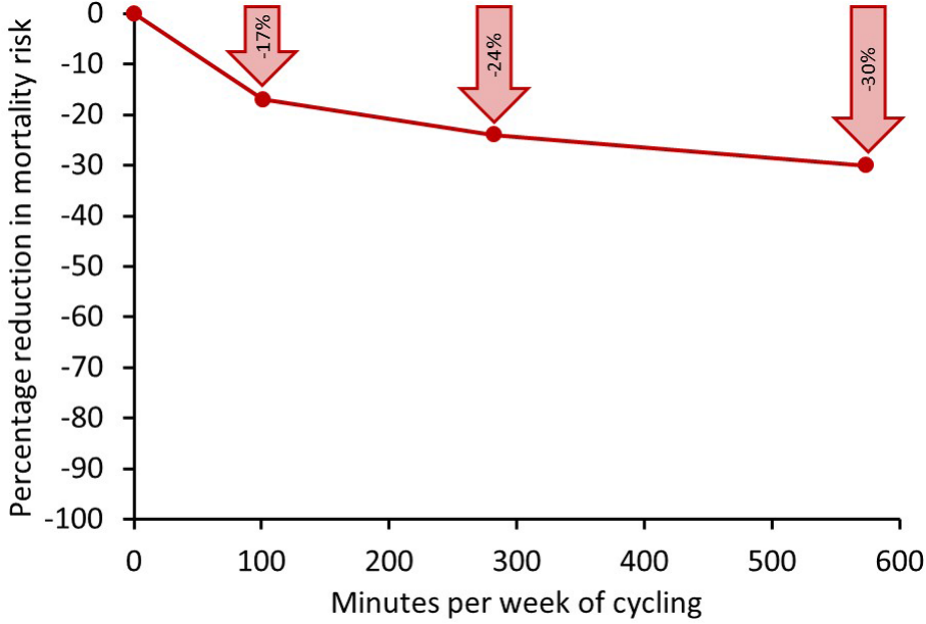
Relative reduction in risk of mortality associated with different weekly volumes of cycling compared with undertaking no regular cycling for a meta-analysis of seven large-scale population studies. Data in individual studies adjusted for a range of confounding factors including age, sex, smoking, other non-cycling physical activity, socio-economic status, and aspects of health status. Points on graph at 100, 280 and 570 min per week represent the mid-points of ranges of 10–210, 210–350, and 350–800 min per week, respectively. Modified from (3).

All but one study reported that higher levels of cycling were associated with a lower risk of mortality over the follow-up period. When data from all studies were combined, there was a clear, statistically significant lower risk of mortality associated with regular cycling participation. The combined data from all of these studies, illustrated in Figure 1, indicated that higher levels of cycling were associated with lower risk of mortality with a curvilinear relationship, with the largest difference in mortality risk being between individuals reporting no cycling and those reporting undertaking up to approximately 100 min per week of cycling. Participation in approximately 100 min of cycling per week was associated with a 17% lower risk of mortality **c**ompared with no cycling participation, in analyses adjusted for major confounding variables.

Levels of cycling beyond this range were associated with a further lowering of mortality risk, but the risk reduction was less steep. Participation in approximately 270 min per week was associated with a 24% lower mortality risk compared with no cycling, however, participation in approximately 570 min per week was associated with a 30% lower risk of mortality compared with no cycling. The clear implication of this is that bigger public health gains will be realised by encouraging individuals who currently do not cycle regularly to do some cycling, rather than getting those who currently cycle regularly to cycle more.

Since the publication of the Kelly et al. (2014) systematic review and meta-analysis (3), further epidemiological studies have examined the association between cycling participation and risk of mortality. Key features of these studies are summarised in Table 1 and described below.

**Table 1:** What is known and what is not yet know about barriers to cycling.

Barriers to cycling		
What is known	What is not yet known	What is needed to fill evidence gap
Key barriers to cycling include:
• Local environment ○ Lack of cycle route/paths ○ Hills ○ Distance to travel ○ Weather • Facilities ○ Lack of secure cycle parking ○ Lack of showers at work (after effortful cycling) • Individual factors ○ Perceived lack of safety ○ Perceived attitude of other road users ○ Convenience of using car ○ Lack of skills ○ Lack of confidence ○ Cost ○ Lack of time due to family, work and social commitments ○ Lack of interest in cycling as a ‘sport’	• Which of the identified barriers to cycling are amenable to change? • Which interventions to reduce barriers are feasible/acceptable/effective? • What are specific barriers to cycling for older adults and how might these be overcome? • What are specific barriers to cycling for different disabilities, and how might these be overcome? • What are specific barriers to cycling for different BAME groups and how might there be overcome? • What are specific barriers to recreational cycling, and how might they be overcome?	• Systematic reviews of the available evidence on barriers to cycling in older adults, specific disabilities and specific BAME groups • Cross-sectional studies on barriers to cycling in these groups • Cross-sectional studies focusing specifically on barriers to recreational cycling • Qualitative studies to examine attitudes and experiences of barriers to overcoming cycling in different population groups and different contexts (e.g. high cycling infrastructure vs. low cycling infrastructure, rural vs. urban, high socioeconomic status vs. low socioeconomic status) • Development and testing of interventions to overcome identified barriers in different populations groups and different contexts.

Koolhaas et al. examined the association between cycling participation and risk of all-cause mortality amongst 7,225 older adults (mean age 70 years) in living in Rotterdam, who were followed up for a median of 13.1 years (6). They reported that participation in “medium” levels of cycling (median 13 min per day, or 91 min per week) was associated with an 28% lower risk of mortality compared with no cycling participation, after adjustment for confounders (Supplementary Table S2), and participation in “high” levels of cycling (median 51 min per day or 357 min per week) was associated with an 35% lower risk of mortality.

To minimise the potential contribution of reverse causality, Koolhaas et al. undertook sensitivity analyses where they excluded deaths occurring in the first 5 and first 10 years of follow up (those with pre-existing disease are more likely to die within the first few years the measurements), and the findings were essentially unchanged (6). This is an important study as it focused on older adults, where there had previously been limited data, and shows that the associations between cycling participation and lower risk of mortality extend into older age. Importantly, the findings are consistent with the Kelly et al. meta-analysis which reported the largest reduction in mortality risk when moving from undertaking no regular cycling to a undertaking approximately 100 min per week with diminishing further returns thereafter (3).

In a large-scale study of 80,306 UK adults followed up for 9.2 years, Oja et al. found that participation in any compared to no cycling was associated with a 15% lower risk of mortality, after adjustment for confounders (7). Interestingly, this analysis revealed no clear effect of self-reported cycling intensity or weekly cycling duration on the association between cycling and mortality, which again is consistent with the findings from the Kelly et al. meta-analysis (3), which indicated that the largest benefit in terms of lowering of mortality risk was observed when moving from no cycling to some cycling.

In the largest study to date of cycling participation and risk of mortality—approximately the size of all previous studies combined—Celis-Morales et al. examined the association between mode of commuting to work and risk of mortality amongst 263,540 participants in the UK Biobank cohort who were followed up for 5 years (8). Compared with non-active commuting (car/motor vehicle or public transport), cycling to work was associated with a 41% lower risk of mortality. Mixed-mode commuting, where participants undertook part of the journey by cycle with the remainder using a non-active form of transport, was associated with a 24% lower risk of mortality. This is an important finding which indicates that for individuals who live too far from work to cycle the whole way could potentially gain substantial benefits from cycling part of the way. There was a dose-response relationship between weekly cycling commuting distance and risk of mortality, with those commuting less than 30 miles per week having 32% lower risk of mortality, compared to non-active commuters, and those cycling more than 30 miles per week to work having a 40% lower mortality risk. Again, this non-linear response with the largest difference in risk seen between no cycling and some cycling, rather than some cycling and more cycling is consistent with the findings of the Kelly et al. meta-analysis (3).

Finally, in a large study of the combined effects of physical activity and air pollution amongst 52,061 older Danish adults living in urban environments, Anderson et al. reported that participation in cycling was associated with a 17% lower risk of mortality and this association was not modified by exposure to high levels of traffic-related air pollution (9).

Thus, in summary, a large body of epidemiological evidence, including data from over half a million participants has consistently demonstrated that participation in regular cycling is associated with lower risk of mortality compared with no cycling. The curvilinear nature of the dose response relationship (Figure 1) indicates that that the largest benefit is seen when moving from no cycling to some cycling. Thus, with a large proportion of the population cycling less than this amount, it is clear that to maximise public health gains, it is necessary to focus on increasing participation amongst those who do not currently cycle regularly, rather than encouraging those who already cycle regularly to cycle more.

### 2.2. Cycling and risk of cardiovascular disease

There is a large body of robust data from prospective cohort studies (approximately 50 studies with a total of over 700,000 participants) that participation in physical activity is associated lower risk of incidence and mortality from cardiovascular disease (CVD) and coronary heart disease (CHD) (10–14), with a curvilinear dose response relationship, similar to that observed for all-cause mortality (Figure 1).

A smaller number of studies have specifically examined the association between cycling participation and incidence or mortality from CVD or CHD (7–9, 15–23). These studies are summarised in Supplementary Table S3. Of the 12 studies identified, including over 700,000 participants, five were from Denmark, four from the UK, and one each from Netherlands, USA and China. Eleven out of 12 studies reported numerically lower risk of CVD or CHD with higher levels cycling of participation (7–9, 15–17, 19–23); in six of these studies this association was statistically significant (8, 9, 16, 17, 22, 23).

Most studies focused on primary prevention and excluded participants with CVD or CHD at baseline. One study considered both primary prevention and secondary prevention data (22), reporting that participation in cycling was associated with lower risk of both a first myocardial infarction (heart attack) and of a subsequent myocardial infarction in those who had already had an event. It seems likely that the lack of a statistically significant association in some of the studies which suggested numerically lower CVD or CHD risk amongst cyclists (7, 15, 19–21 )may reflect insufficient statistical power to robustly detect an association, rather than the absence of benefit. However, the directional consistency of the findings suggests that if a meta-analysis was undertaken combining all the available data, an overall statistically significant association between cycling participation and lower risk of CVD and CHD would likely be observed.

### 2.3. Cycling and risk of cancer

Studies which have considered the association between cycling specifically and cancer incidence and mortality amongst populations who did not have cancer at baseline are summarised in Supplementary Table S4. Studies examining the association between physical activity and survival from cancer in cancer patients were not considered. Nine studies were identified (8, 9, 15, 16, 19, 20, 24–26) investigating the association between cycling participation and cancer. Two studies reported that participation in cycling was associated with lower risk of overall cancer incidence and mortality (8, 20), but others did not observe a statistically significant association between cycling participation and cancer risk (9, 15, 16, 19). Cycling was associated with lower risk of lung cancer in women but not in men (26), but was not associated with significantly lower risk of colon cancer (24) or prostate cancer (25). Thus, the available evidence on cycling and risk of cancer is mixed and further larger studies with sufficient statistical power to robustly assess effects of cycling on incidence of specific cancers are needed.

### 2.4. Cycling and risk of type 2 diabetes

A number of cross-sectional studies have indicated that participation in cycling is associated lower prevalence of type 2 diabetes (8, 27–30), but relatively few prospective cohort studies have investigated whether cycling participation amongst those who are free from type 2 diabetes at baseline is associated with lower incidence of developing the disease. These studies are summarised in Supplementary Table S5.

Rasmussen et al. (31) evaluated the effects of cycling participation on incidence of type 2 diabetes in 52,513 Danish adults aged 50–64 years at baseline and followed up for 14.2 years. Cycling participation was assessed by self-report questionnaire at baseline, then again approximately 5 years later. This study showed that increasing levels in both total cycling and commuting cycling were associated with lower risk of type 2 diabetes in a curvilinear dose-dependent manner with the greatest benefit seen when comparing those participating no cycling to some cycling, analogous to the relationship seen between cycling and all-cause mortality (see Figure 1). It also observed that individuals who did not cycle at baseline, but had initiated cycling by the second assessment had a 20% lower risk of developing type 2 diabetes than those who were consistently non-cyclists, showing that initiation of cycling even in mid-to-late adulthood was associated with benefit. Interestingly, year-round cycling was associated with lower risk of type 2 diabetes than cycling in summer or winter only, emphasising the importance of cycling consistently throughout the year for maximal risk lowering.

An earlier study of 70,658 Chinese women aged 40–70 years followed up for 14.6 years by Villegas et al. (32) reported that participation in cycling was associated with a 19% lower risk of developing type 2 diabetes, but this was attenuated to a 14% lower risk in a sensitivity analysis which excluded participants with coronary heart disease, stroke, cancer at baseline.

Hu et al. (33 )undertook an analysis of 70,102 US Nurses aged 40–65 years at baseline and followed-up for 8 years, finding that participation in any cycling was associated with a non-significant 4% lower risk of type 2 diabetes. However, it is important to note that this analysis used relatively crude measure of cycling participation (any vs. none) and the reported risk estimate was after adjustment for all other physical activities which were more robustly quantified than cycling participation.

The wider body of evidence evaluating the association between overall physical activity and type 2 diabetes risk is also consistent the available data on cycling. In 2015, Aune et al. (34 )performed a systematic review and meta-analysis of studies investigating the association between physical activity and risk of type 2 diabetes, reporting that participation in 5 h per week of leisure-time physical activity was associated with a 25% lower risk of incident type 2 diabetes than no leisure-time physical activity participation, with the shape of the dose-response curve being curvilinear, such that the largest differences in risk were observed when comparing those undertaking approximately 1–3 h per week of leisure-time physical activity with those undertaking none.

Thus, the available evidence for cycling participation and risk of type 2 diabetes enables broadly similar conclusions to be drawn as the data for cycling and risk of mortality. Cycling is associated with lower risk of type 2 diabetes and substantial differences in risk are seen when comparing individuals who undertake some, compared to those who undertake no regular cycling. Approaches to encourage individuals who currently do not cycle regularly to do some cycling is likely to lead to substantial public health gains.

### 2.5. Cycling and risk of obesity

Cross-sectional studies generally report that individuals who participate in cycling have a lower body mass index (BMI), and a lower prevalence of overweight and obesity, than their non-cycling counterparts (27, 28, 35–39). Relatively few prospective studies have evaluated the association between participation in cycling and future risk of overweight or obesity or future changes in body weight. These studies are summarised in Supplementary Table S6. Taken together these studies suggest that participation in cycling has a modest association on change in weight or BMI (<1 kg difference compared with not cycling), waist circumference (∼0.5 cm difference) and incidence of obesity (∼15%–25% lower risk of developing obesity) (31, 40–42). This is consistent with analyses of the association of overall physical activity and changes in weight and waist circumference (43). While these differences may be small on an individual level, at a population level they may be sufficient to elicit a public health gain, suggesting that cycling could contribute to public health strategies for obesity prevention.

### 2.6. Cycling and bone health

Physical activities which are weight-bearing and induce substantial skeletal impacts are associated with benefits on bone health, including higher levels of bone mineral density (44–46) which can potentially lead to lower risk of osteoporosis and fracture particularly in older adults (46–48), but as cycling is non-weight bearing form of physical activity, it is not possible to simply extrapolate evidence from other forms of activity to cycling.

Olmedillas et al. published a systematic review of the evidence on cycling and bone health (49). The majority of included studies were of high-level competitive cyclists, and suggested that cycling at this level does not generally appear to have a beneficial effect on bone mineral density, and may be associated with low bone mass in some cases (49).

One study included in the review reported that sprint cyclists had stronger bones than longer-distance cyclists (50); another reported that cross-country mountain bikers had higher bone mineral density than road cyclists (51), which is consistent with the higher mechanical loading to bone experienced during sprint cycling and mountain biking compared to endurance road cycling.

There is an absence of data in the literature on the effects of participation in transport and recreational cycling on aspects of bone health. Such studies are needed but given that high level competitive cycling is associated with neutral or negative effects on bone health, it seems unlikely that recreational or transport cycling would have a substantial positive effect in this domain. Thus, particularly for people at risk of osteoporosis (for example women and older adults), the available evidence suggests that cycling should probably not be the sole form of physical activity undertaken and should ideally be supplemented with other forms of bone-strengthening weight-bearing physical activities to maximise bone health. A time-efficient way to do this might be to perform exercises such as vertical jumps (e.g., ∼50 jumps per session in sets of 10–20) a few times per week (46).

### 2.7. Evidence from intervention studies on cycling and health outcomes

A combination of observational and intervention studies is needed to gain a complete picture of how cycling participation affects health outcomes. However, studies evaluating the effects of cycling interventions specifically on health outcomes are limited. In 2011, Oja et al. (52) undertook a systematic review of studies evaluating health benefits of cycling (excluding studies of stationary cycling on a cycle ergometer) and identified four intervention trials (53–56). These studies and other cycling intervention studies evaluating health outcomes of real-world cycling interventions (as opposed to intervention using stationary cycle ergometers) published since publication of this systematic review (57, 58) are described in Supplementary Table S7.

The overall picture is that the evidence-base for cycling interventions and health outcomes is very limited, with the published studies all being small (less than 100 participants) and generally short-term. The available evidence indicates that cycling interventions improve cardiorespiratory fitness. There is no clear evidence which indicates effects on body composition or on biomarkers of chronic disease risk, which is likely due to the studies being too small to have sufficient statistical power on these health outcomes. The long-term effects (>1 year) of cycling interventions on *any* health outcomes are also not known at present. Thus, adequately powered larger, longer-term studies are urgently needed to establish the extent to which feasible large-scale cycling interventions are likely to alter health outcomes in real-world settings.

### 2.8. Summary of evidence on cycling and physical health outcomes

There a large body of observational data indicating an association between participation in cycling and favourable health outcomes, with the evidence suggesting the largest potential public health gains are likely be realised by encouraging individuals who currently do not cycle regularly to do some cycling, rather than getting those who currently cycle regularly to cycle more. The evidence also indicates that the benefits of cycling are broadly similar to other forms of physical activity, thus to maximise gains in public health, it would be important to increase participation in cycling amongst individuals who currently undertake little physical activity, rather than encouraging those who are currently active in other pursuits to switch to cycling. However, the evidence from intervention trials is much more limited. Thus, there is a clear need for longer, larger RCTs to evaluate the size of potential health gains which are realistically achievable from pragmatic real-world interventions to promote cycling. Table 2 provides a summary of what is known and what is not yet know about cycling and physical health.

**Table 2:** What is known and what is not yet know about the benefits and risks of cycling.

	What is known	What is not yet known	What is needed to fill evidence gap
Cycling and physical health outcomes	• A large body of data from prospective cohort studies indicates that participation in cycling is associated with lower risk of mortality from any cause, cardiovascular disease and type 2 diabetes. • The dose-response relationship is curvilinear with the largest lowering of risks seen when comparing no cycling with moderate levels of cycling. Thus, the largest potential for health gains likely to come from increasing participation amongst those who do not currently cycling regularly, rather than encouraging those who already cycle regularly to cycle more. • Evidence from prospective cohort studies for an association of cycling participation and cancer incidence and mortality is more mixed, with one major study showing lower cancer risk amongst regular cyclists, but other studies showing no significant association. This may reflect, in part, insufficient statistical power to detect any such associations. • In prospective cohort studies, there is evidence of a modest association between cycling participation and lower bodyweight (<1 kg difference compared with not cycling) and waist circumference (by ∼0.5 cm). These differences are relatively small on an individual level but may at a population level be sufficient to elicit a public health gain. • The association between cycling and bone health appears to be neutral at best, and may be negative for high level competitive cycling. Thus, particularly for people at risk of osteoporosis (for example women and older adults), the available evidence suggests that cycling should probably not be the sole form of physical activity undertaken, and should ideally be supplemented with other forms of bone-strengthening weight-bearing physical activities to maximise bone health. Evidence from a small number of intervention trials indicates that increasing cycle commuting leads to improvements in cardiovascular fitness, but effects on body weight or on biomarkers of chronic disease risk are unclear.	• The observational nature of the majority of the available data makes difficult to draw firm conclusions about the extent to which the associations between cycling and health outcomes are causal, or the extent to which pragmatic, real-world interventions to increase cycling are likely to induce a sustained impact on health outcomes (or biomarkers of risk for such health outcomes). • Effects of cycling interventions on health outcomes other than cardiorespiratory fitness are currently unclear, as studies have generally been small, short-term, with insufficient statistical power to detect such effects.	• Large-scale, long-term (at least 1 year duration, ideally longer) interventions trials (ideally randomised) are needed to evaluate the sustained effects of increasing cycling participation amongst those who don't currently cycle regularly on health biomarkers causally related to risk of chronic disease (for example, adiposity, blood pressure, blood lipids, glucose, insulin). • Such trials would ideally encompass a wide range of demographic groups. • Such trials will provide evidence of a causal relationship between cycling participation and health outcomes and enable quantification of the extent to which health outcomes can be realistically altered by increasing cycling participation. This is vital evidence for public health decision makers.
Cycling and mental health, quality of life and wellbeing	• In cross-sectional studies, participation in cycling is associated with lower levels of perceived stress, higher levels of commuting enjoyment, better perceived general health and higher perceived quality of life. This is consistent with the wider evidence base on physical activity in general and mental health, quality of life and wellbeing outcomes. • Cross-sectional and prospective cohort studies suggest that cycle commuting is associated with less sickness absence amongst employees	• It is not known whether associations between cycling and favourable mental health, quality of life and wellbeing are causal. From the available evidence it is not possible to fully exclude the possibility that those with better perceived health or quality of life may be those who choose to cycle more, rather than cycling leading to improvements in these health outcomes. • It is not known the extent to which pragmatic, large-scale interventions to increase cycling amongst those who do not cycle regularly can produce sustained improvements in mental health, quality of life and wellbeing outcomes.	• Intervention trials (ideally randomised) are needed to evaluate the effects of increasing cycling participation amongst those who don't currently cycle regularly on these outcomes. • Such trials would ideally be large, encompass a wide range of demographic groups, and long enough to evaluate sustained effects (ideally at least 12 months). These trials will also enable quantification of the extent to which these outcomes can be altered by increasing cycling participation.
Risks of cycling associated with collisions and exposure to air pollution	Collisions • Risks of collisions with cycling are somewhat higher than driving, but still very low in absolute terms • The health benefits of cycling are more than 25 times greater than the slightly increased risk of collision risk • Collision risks are lower where there is good cycling infrastructure and providing physical separation between bicycles and motor vehicles improves safety Air pollution • Higher ventilation rates during cycling mean inhalation of air pollution particles can be higher during cycling than driving, but pollution gradients are steep on and near roadways so small changes in position on the road relative to vehicles can have substantial effects on exposure • Using cycle lanes and lower traffic routes can reduce exposure to air pollution for cyclists • The health benefits of cycling substantially outweigh the potential risks of increased exposure to air pollution in all but the most extreme air pollution conditions worldwide. Even in Delhi (the most polluted city on the WHO database) undertaking up to 45 min of cycling per day was estimated to provide a net health benefit.	• Collision risks of cycling compared with driving when true like-for-like comparisons are made (for example excluding motorway driving, and off-road cycling such as mountain biking and BMX) • Risks of cycling in polluted environments for susceptible populations, for example those with respiratory problems are uncertain • Limitations in modelling risks associated with pollution, for example by only considering long-term average exposure levels, add uncertainty to the risk estimates	• Further studies making better like-for-like comparisons of collisions associated with cycling and other modes of transport • Studies examining the risks of cycling at different levels of air pollution in populations who may be more susceptible to adverse consequences of high levels of air pollution • More sophisticated studies modelling the impact of pollution exposure during cycling on health outcomes, taking into consideration varying exposures to air pollution
Economic benefits and costs of cycling	• Replacing car journeys with cycling journeys is likely to be cost saving to the individual. Limited data suggest that overall cost per km travelled, taking into account direct financial outlays, costs of time, health benefits and risks of collisions for cycling is less than half that for car journeys. • Limited data suggests that increasing cycling amongst employees is likely to be cost saving to employers in terms of lower absenteeism costs. • Cycling generate has the potential to substantial economic benefits to related to health. For example, economic modelling studies have projected that: • Increasing cycling to 25% of all journeys in the UK by 2050 would provide over £42 billion in economic benefit (including £35.5 billion due to personal health gains). • Increasing cycling by 3 km per day and walking by 1 km amongst individuals in urban centres in England and Wales would result in £17 billion in savings to the NHS over 20 years • Replacing car journeys with cycling would result in economic benefits in terms of lower pollution and emissions • Losses related to reduced duty and taxation associated with replacing car journeys with cycling are more than an order of magnitude lower than the benefits. It may be cost effective to society to subsidise cycling to increase uptake. • In 2011, cycling contributed an estimated £2.9 billion to the UK economy • Investing in cycling infrastructure creates more jobs than the same level of investment for other transport infrastructure	• Economic models of cost-benefit of replacing car journeys with cycling for the individual have only been undertaken in limited settings, and do not currently include key aspects such as personal valuation of safety, potential discomfort and other costs such as insurance. • Estimates of absenteeism cost savings to employers associated with cycling amongst employees are based on observational estimates in a limited range of occupations, and the extent to which interventions to increase cycling participation would change absenteeism rates are not known. The effects of cycling amongst employees on productivity outcomes other than absenteeism is also not known. • RCTs which capture the full cost savings to an individual (and employer) and their value of time have not been conducted. This would inform the most worthwhile investment of new cycling initiatives. • Methodologies such a cost-benefit analyses, conducted from a societal perspective are challenging to conduct and to date few have been fully societal in their assessment of benefits. • From a behavioural economics perspective, we don't know which attributes predict changes in cycling behaviour of the adult population and what the trade-offs are. For example, are people willing to commute for a longer time to increase their personal health benefits? Are people willing to accept financial incentives to increase their cycling level? Is there a gender balance effect on cycling levels and if so, what do women/men need to incentivise them to increase cycling activity?	• Large-scale, long-term interventions which capture all resource use and costs relating to an individual's cycling behaviours and their health-state would facilitate a more accurate understanding of costs and possible associated benefits. • Societal cost-benefit analyses detailing the costs and benefits across multiple sectors such as health care, employment, transport, retail and education would generate the full societal picture of increasing cycling. • Evidence is needed on the attributes (and levels) which predict cycling uptake for women and men along with evidence on the trade-offs between attributes such that initiatives can be optimally designed tailed to increase cycling.

## 3. The benefits of cycling on mental health, quality of life, and wellbeing outcomes

A large body of evidence suggests that physical activity in general is associated with a range of positive mental health and wellbeing outcomes, including increased sleep quality, improved executive function and other components of cognition, reduced risk of depression and depressive symptoms, and higher perceived quality of life (59, 60). However, there is more limited evidence on outdoor cycling specifically and mental health, quality or life and wellbeing outcomes. Key studies on this topic are summarised in Supplementary Table S8.

A consistent body of evidence from cross-sectional studies suggests an association between cycling and aspects of mental health, quality or life and wellbeing outcomes. For example, cross-sectional studies have reported associations between cycling and lower levels of perceived stress (61, 62), higher levels of commuting enjoyment (63), better perceived general health (64, 65), high levels of quality of life (66, 67), and higher life satisfaction (68). Evidence from cross-sectional (69) and prospective cohort (70) studies have also indicated that cycle commuters have less sickness absence (by∼1 day per year) compared with those who commute to work by other means.

Intervention trial data are more limited. One non-randomised intervention trial indicated that an intervention increasing commuting cycling led to greater vitality at 6 months, but this was not sustained until 1 year (55). Thus, the available evidence for cycling and health, quality of life and wellbeing outcomes, while relatively limited, is consistent with the evidence base for overall physical activity which indicates benefits for these outcomes.

There is a clear lack of trial data about whether interventions to increase cycling will improve mental health, quality of life and wellbeing. Randomised controlled trials to quantify the effect of pragmatic and feasible cycling interventions on these outcomes are urgently needed. In particular, understanding whether cycling interventions can reduce sickness absence amongst employee could potentially play a key role in encouraging employers to introduce interventions and policies which facilitate active commuting. Table 2 summarises what we know and what we do not yet know about cycling and mental health, quality of life and wellbeing.

## 4. The risks of collisions and exposure to air pollution for cyclists

### 4.1. Cycling and risk of collisions

Collision rates in cycling can be expressed based on a number of different exposure measures, including collisions per trip, per unit distance, or per unit time, amongst others (4). The most appropriate measure will depend on the context—collision risk of cycling in comparison to cars is higher when expressed per unit distance than per unit time or per trip as cars travel much faster and further than cycles for any given trip (4, 71). This has led some cycling advocates to suggest that a distance-based comparison of cycling and car safety is not appropriate unless only short-distance car journeys which could be feasibly replaced by cycling are compared, and long-distance car travel (which is an order of magnitude less dangerous than local driving) is excluded (4, 71).

**Figure 2:**
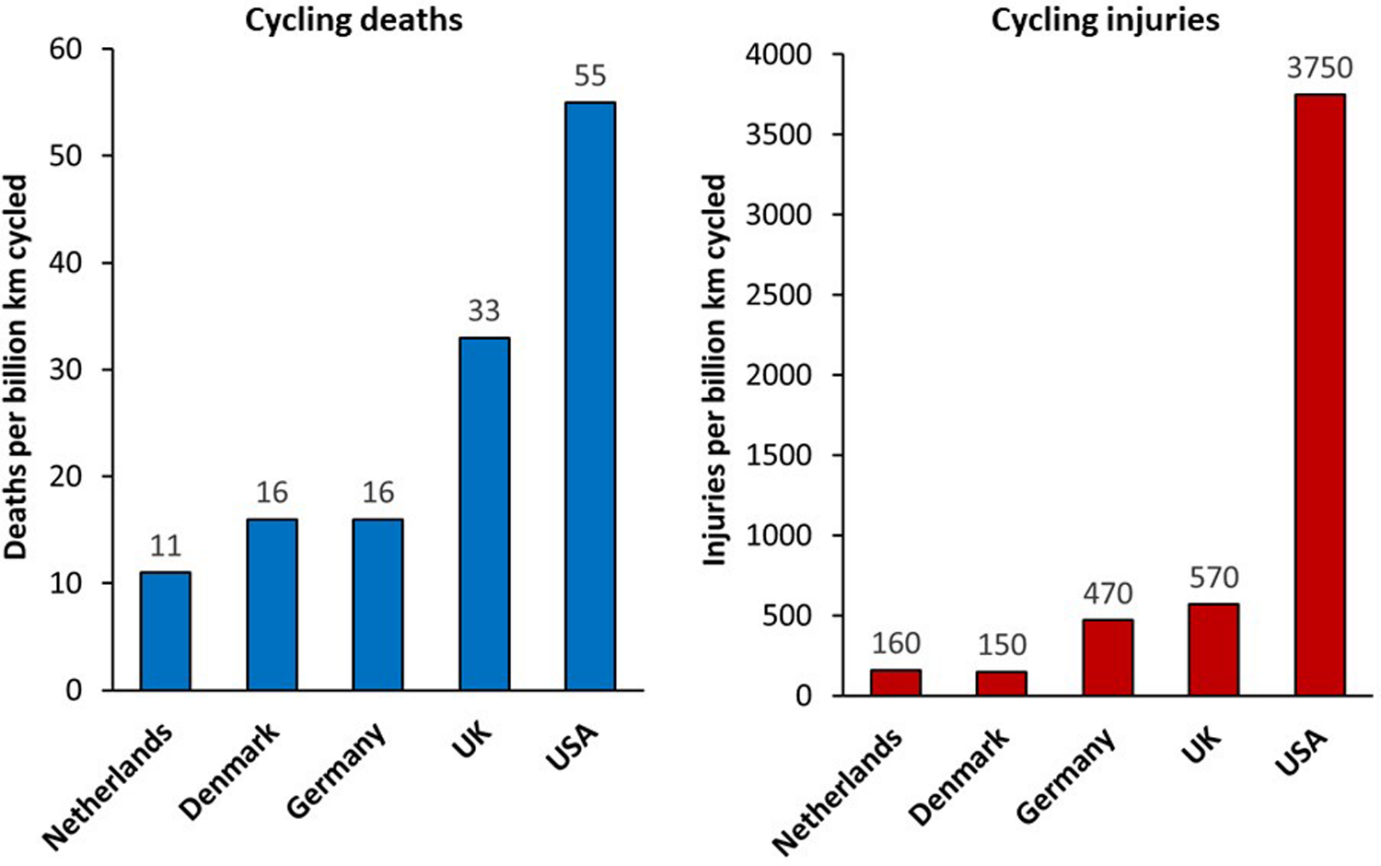
Rates of cycling deaths and non-fatal injuries in selected countries in 2004–2005. From (72).

Figure 2 shows rates of cycling fatalities and injuries for cyclists, expressed per unit distance, from selected countries in 2004 to 2005 taken from a 2008 review by Pucher and Buehler (72). This indicates that absolute rates of injuries are low, and fatalities are very rare events, however these are lowest in countries with high levels of cycling infrastructure.

Mindell et al. (71) reported hospital admissions and mortality data in England in 2007–2009 for cyclists, pedestrians and car/van drivers by age and sex groups, revealing that all-age fatalities varied within the same factor-of-three range for men (0.15–0.45 fatalities per million hours' use) and women (0.09–0.31 fatalities per million hours' use) across these three transport modes, but with substantial variation by age and sex, with a higher fatality risk for driving compared with cycling for young men, and relatively higher risks for cycling mortality in men aged over 70 years. Risks of hospital admission from cycling (29 admissions per million hours' use in men and 28 admissions per million hours' use in women) were higher than for driving (1.6 admissions per million hours' use in men and 1.8 admissions per million hours' use in women), but for cycling this does include admissions due to activities such as mountain biking and BMX, as well as cycling for transport, so does not provide a true like-for-like comparison with driving (71).

Risks of cycling vary according to route infrastructure with lower probabilities for a crash being evident on protected bike lanes (by ∼30%–90%) than on roads shared with motorised traffic (73, 74). A review by Reynolds et al. concluded that clearly marked bike-specific facilities were consistently shown to improve safety for cyclists, reducing injury or crash rates by about half compared to unmodified roads (75).

Overall, absolute risk of collisions with cycling are somewhat higher than driving, but still very low in absolute terms, and the health benefits of cycling are more than 21 times greater than any increased risk of collision (76). In short, even with current levels of cycling collision rates, the benefits of cycling substantially outweigh any increased collision risk. However, there is clear potential to reduce the collision risk associated with cycling further. Risks of cycling are lower in countries with good cycling infrastructure (71, 72, 77), and there is clear evidence that improving cycling infrastructure and providing physical separation between bicycles and motor vehicles improves safety (78).

Modelling the impact of interventions on cycling collision risks is complex and findings are difficult to generalise, but the available evidence indicates that increases in cycling lead to disproportionately smaller increases in collisions and that the negative impact of collisions do not outweigh the benefits of physical activity from cycling (78, 79).

### 4.2. Cycling and risk of exposure to air pollution

This section considers the risks of exposure to air pollution to the individual cyclist while they are cycling. Exposure to air pollution tends to be higher when travelling on roads than during most other activities due to proximity to motor vehicles (80). This is particularly in urban environments with high vehicle density, during peak commuting hours and exposure to pollution during commuting makes a substantial contribution to total personal exposure to air pollutants (81).

Karanasiou et al. (81) undertook a review of levels of personal exposure to key air pollutants during different modes of commuting. Twenty European studies were identified which calculated exposure to air pollution during cycling, which reported exposures for PM2.5 in the range 29–72 µg/m^3^, P10 in the range 37–62 µg/m^3^, black carbon in range 3–21 µg/m^3^ (81). These exposure levels were broadly similar to those in cars (P2.5 22–85 µg/m^3^, P10 36–76 µg/m^3^, black carbon 6–30 µg/m^3^) (81). When the higher ventilation rates, and potentially longer travel times, for cyclists are taken into account, inhaled doses of particles can be up to 4–7 times higher than car passengers on the same route (80, 81).

A key issue is whether adverse effects of exposure to pollution negates health benefits of cycling when cycling in areas with elevated levels of air pollution. The available evidence suggests that this is not the case. In a prospective cohort study of over 52,000 Danish adults aged 50–65 years, followed up for 13 years, reductions in risk of disease specific (cardiovascular disease, respiratory disease, diabetes) and all-cause mortality associated with cycling were not modified by level of exposure to traffic-related air pollution (assessed as NO_2_) exposure (22).

Modelling studies of the effects of cycling on health outcomes also estimate that the health benefits associated with increased physical activity from cycling are several fold higher than the risks associated with increased inhaled air pollution doses. Mueller et al. (82) reported that health impacts related to differences in air pollution exposure were small, and the benefits associated with increased physical activity associated with taking up cycling were appromately an order of magnitude greater than the risks to the cyclist assocated with increased inhaled air pollution.

Tainio et al. (83), extended this work by modelling the risk-benefit balance of cycling in environments with increasingly high levels of air pollution. In this analysis they calculated a “tipping point” for different levels of air pollution where maximal health benefits are achieved and increasing cycling beyond this level would not accrue further health benefits, and a “breakeven point” where cycling beyond this level would lead to the risks exceeding the benefits. For cities with average the global average level of urban background pollution in the World Health Organistation database (PM2.5 levels of 22 µg/m^3^; for context the level in London is lower than this at 16 µg/m^3^), the tipping point would only be reached after 7 h per day of cycling and breakeven point would never be reached. For the average European city the tipping point was 2.5 h per day of cycling and breakeven point was 9.25 h of cycling. Even in the most polluted city in the database (Delhi, background PM2.5 levels of 153 µg/m^3^) the tipping point was 30 min of cycling per day and breakeven point was 45 min of cycling per day. Thus, the evidence indicates that in almost all but the most extreme air pollution conditions worldwide, the benefits of cycling exceed the risks of increased exposure to inhaled air pollution for all reasonable durations of daily cycling.

A summary of what we know and is not yet know about cycling and risk of collisions and exposure to air pollution is shown in Table 2.

## 5. The economic benefits and costs of cycling

The following section discusses the available evidence and current research gaps on the economic benefits and costs associated with cycling, considering costs to the individual, employers and wider society. We searched the academic and grey literature for reports on the economic benefits and costs of cycling, prioritising reporting data from reviews summarising existing evidence, but also reporting data from individual studies. Thus, this section provides a representative overview of the existing evidence, but does not represent a systematic review of the topic.

When evaluating the economic costs and benefits of cycling (in comparison to other modes of transport), it is important to consider costs at multiple levels. These include costs and benefits:

• To the individual (such as value of time, cost of purchase and depreciation, cost of fuel, health).
• To employers (such as productivity, absenteeism).
• To wider society (such as health and social care, congestion, environmental impact, collisions, taxation, employment).

Costs and benefits of cycling at these three levels are described in the sections below.

### 5.1. Economic benefits and costs of cycling to the individual

Evidence from Denmark indicates that choosing to cycle over driving a car is cost saving to the individual (84). Taking into account costs from vehicle taxes, fuel, oil, tires, repairs and depreciation, it was estimated that cycling one kilometre costs the individual €0.048 (£0.043), whereas travelling the same distance by car would cost €0.34 (£0.29) (at 2008 prices, currency exchange rates correct as of 06/12/2022 from www.xe.com). If taxation costs (which can vary considerably by country and vehicle emissions) are excluded, the cost of travel was per km €0.039/km (£0.035) by cycle and €0.16 (£0.14) by car.

As cycling is generally slower than driving—although this is not necessarily the case for urban commutes—cost of time is the highest cycling-related cost. Time costs valuations are derived from estimates of a population's willingness to pay for time changes in length of travel time and differ according to mode of transport (car, cycling or public transport)(84). It also differs between societies, so care must be taken in extrapolating Danish time cost data elsewhere. The Danish study assumed cycling speeds of 16 km.h^−1^ and driving speeds of 50 km.h^−1^, and costed driving time at €15.19/hour and €22.86/hour for delays (people as less tolerant of time in traffic, so time is costed higher for delays) and costing cycling time at €12.10/hour and €18.28/hour for delays, equating to a time cost of €0.672/km for cycling and €0.215/km (84).

The Danish reports estimated that cost to the individual of cycling collisions at €0.034/km (vs. €0/km for driving, though driving collisions did incur social costs, see Section 6.3 below), but that cycling was associated with individual gains in terms of prolonged life (by €0.358/km) and improved health (by €0.149/km) (84). Taken all of these factors into account the cost to the individual was €0.243/km for cycling and €0.511/km for travelling by car (84). Thus, overall these data suggest that travelling by cycling, even after accounting for additional time taken, reduces the economic costs of commuting by more than half, but further such economic evaluations in other contexts are needed extrapolate findings beyond a Danish setting. For example, in many urban contexts in the UK, assuming a driving speed of 50 km.h^−1^ is optimistic. Furthermore costs associated with insurance, as well as costs associated with the personal valuation of perceptions of safely (which may increase cycling costs) and potential discomfort during travel were not considered in this economic model. Inclusion of such factors is needed to provide a more complete picture of economic costs and benefits to the individual.

### 5.2. Cycle commuting and productivity

There is limited evidence available on the effects of cycle commuting on productivity. Data from studies in the UK and Denmark both reported that regular cyclists are more likely to have lower absenteeism per year than their non-cycling counterparts, by approximately 1 day per year (69, 70). A 2007 report by SQW for Cycling England on valuing the benefits of cycling, assumed that increased physical activity from cycling would lead to 0.4 days less absence per year, resulting in £64.40 saving per working person per year [assuming gross value added (GVA) per employee of £37,000 per year] (85).

Data on the cost-benefit of work-based cycle schemes is also limited. A 2008 report from Transport for London summarising the current state of knowledge on cycling in London to inform cycling policy, which reviewed evidence from over 100 studies, identified a work-based cycle pool scheme called Bikes for Business which could reportedly provide savings of £25–80/month per bike to the employer (due to reduced taxi and public transport costs) and approximately £50/month to the individual employee due to reduced travel costs (86). Furthermore, qualitative research reported that this scheme reduced employee travel time and avoided parking problems and was viewed by some employees as directly improving their productivity.

Thus, while the available data on cycling and productivity are limited the current evidence suggests a potential net benefit to employers of higher levels of cycling amongst employees. Development of employer-led interventions to increase levels of cycling amongst employees could conceivably result in net economic benefit to employers, even when costs of the intervention are taken into consideration.

Further research is needed to more robustly quantify the benefits and costs to employers, as well as employees, of pragmatic, large-scale interventions to increase cycling amongst employees. The first stage in this process would be to gain a better understanding of which type of interventions are most likely to be cost-effective in incentivising employees to increase their cycling participation.

### 5.3. The economic benefits and costs of cycling to wider society

There is a growing body of evidence indicating a net benefit to society of increasing participation in cycling as summarized in the following sections relating to cycling impacts on health, pollution and congestion, duty and taxation and employment and retail.

#### 5.3.1. Economic costs and benefits of cycling in relation to health

To assess the economic costs and benefits of cycling in relation to health, it is necessary to first obtain an estimate of the epidemiological dose-response relationships between cycling and health benefits and health risks, such as pollution exposure and collisions, as well as potential changes to pollution as a result of changes in cycling and then scale them into an impact model according to level or change in level of population cycling (4). These health effects can be expressed in a number of ways. Typically, economists use incidences of chronic diseases prevented, disability adjusted life years (DALYS), premature mortality, years of life lost, or years of healthy life lost), and by placing an economic value on these health outcomes (for example, £30,000 per DALY avoided).

Commonly used models relevant to this report include the World Health Organization's Health Economic Assessment Tool (HEAT) for cycling and walking (87, 88) and the Integrated Transport and Health Impact Modelling Tool (ITHIM) (89). All such models assume that any cycling represents additional physical activity, rather than displacing other forms of physical activity, and also make assumptions about the nature of the dose-response relationship between cycling and health which are not fully understood, for example about benefits of very short duration bike rides and the tradeoff between intensity and duration for benefits (4). Some models attempt to take into consideration differential benefits according to other factors such as age. The models also assume that the epidemiological association between cycling and health outcomes is entirely causal and the level of risk reductions seen with increasing levels of cycling in observational studies would be replicated by interventions to increase cycling, which may not necessarily be the case. Notwithstanding these limitations, several studies have been undertaken to estimate the economic value of potential effects of cycling on health. These consistently report net health benefits of cycling would result in cost savings to national health budgets.

In 2008, Cavill et al. systematically reviewed economic analyses of transport infrastructure and policies which included health effects related to cycling (90). This review, which pre-dated the introduction of the HEAT and ITHIM modelling tools, included 15 studies, 10 of which considered only cycling and five considered cycling and walking, all but one of these studies (which was from the USA) were conducted in European countries. Three studies were considered high quality, five moderate quality and seven low quality in terms of methodology. While the studies were heterogeneous in nature and presented a wide range of results, benefit-cost ratios (present value of benefits/present value of costs) and the value attributed to each new cyclist were frequently reported outcomes. All but one study reported a positive benefit-cost ratio, with a median benefit-cost ratio of 5:1 and a range from −0.4 to 32.5. However, the authors suggested that these values should be treated with caution given the different assumptions made across studies. Six studies presented results in terms of value attributed to each new cyclist ranging from €127 to €1,290, with much of the difference in value due to different assumptions [some which Cavill et al. (90) considered incorrect], and many studies not being transparent in their methods. However, these values are in line with a report by SQW Consulting for Cycling for England which estimated the monetary value of one additional cyclist cycling for one year, replacing 50% of car trips with cycling trips. These models calculated benefits of £408.67 per cyclist per year in terms of lower risk of loss of life with an additional £28.30 per year in NHS savings (91).

Some reports have attempted to quantify population-level health cost savings associated increasing levels of cycling, or active travel. Jarrett et al. (92) modelled the effects on NHS costs (including management and treatment of acute and chronic diseases, and road traffic injuries) over 20 years of increasing cycling by 3 km per day and walking by 1 km per day, amongst all individuals in England and Wales living in urban areas (those with 20,000 residents or more). The model estimated a saving of ∼£17 billion over 20 years to the NHS budget (at 2,010 prices)—approximately 1% of the yearly health care budget.

A report by Fishman and coworkers (93), used HEAT to estimate the economic health benefits of cycling in the Dutch population. Based on age-specific weekly cycling times (average of 74 min per week across the age range from 20 to 90 years) and mortality rates it was estimated that current levels of cycling prevented 6,657 deaths per year in the Netherlands, resulting in a 0.57 year increase in life expectancy and an annual economic benefit of €18.6 billion, assuming a value of a statistical life of €2.8 million per prevented death.

#### 5.3.2. Economic benefits and costs of cycling in relation to pollution and congestion

The available evidence, albeit limited, quantifying the benefits of cycling on congestion and pollution consistently reports that increased cycling levels would lead to reductions in associated costs. These approaches model the effects of replacing car journeys with cycling journeys and monetary attribute costs to reductions in emissions, noise and congestion. Using this approach it was estimated that, in Copenhagen, replacing car travel by cycling would result in a saving of €0.004/km in air pollution costs, €0.005/km in climate change costs, €0.0062/km in congestion costs (84, 94).

Crawford and Lovelace (95) used Transport Analysis Guidance (TAG) data from the Department for Transport to estimate pollution and congestion cost savings which could be realised by meeting CDP and GBC cycling targets in 2025 and 2050. Compared with a baseline of zero growth in cycling:

• The CDP plan was projected to result economic benefits in 2025 of £283.5 million in reduced congestion, £10.9 million in reduced greenhouse gas emissions, £2.4 million in decreased noise and £1.5 million in improved air quality. Corresponding economic benefits in 2050 were £956.3 million (reduced congestion), £36.6 million (greenhouse gases), £7.9 million (noise) and £5.2 million (air quality).
• The more ambitious GBC target was projected to result in economic benefits for congestion, greenhouse gases, noise, and air quality of £1.09 billion, £41.5 million, £9.1 million, and £5.9 million, respectively in 2025; and £7.1 billion, £271.6 million, £59.2 million, and £38.6 million, respectively in 2050.

Modelling studies evaluating potential effects of increasing levels of cycling for transport on air pollution (Table 2) also considered the effects on costs associated with congestion. Creutzig and coworkers (96) modelled the effects of policy scenarios to reduce motorised transport use and increase use of public transport and non-motorized transport, which including increasing cycling infrastructure, on pollution and congestion in four European cities. Economic values for pollution changes were not reported, but it was estimated that transport scenarios in Malmo and Freiburg which resulted in increases in non-motorised transport trips by ∼50% would provide estimated economic benefits of reduced congestion of €363 million per annum (∼€800 per person per annum) and €184 million per annum (∼€650 per person per annum) respectively in the two cities.

## 6. The barriers to cycling participation

A detailed understanding of barriers to cycling amongst those who do not cycle regularly is essential to inform the design of effective interventions to facilitate increased levels of cycling. This section discusses the evidence on barriers to increasing cycling in adults. This evidence comes from two sources. First, peer-reviewed studies were identified via searches of key databases [PubMed, CINAHL (Cumulative Index to Nursing and Allied Health), AMED (Allied and Complimentary medicine database), PeDRO (Physiotherapy Evidence Database), Cochrane library]. Although our search was not exhaustive, it is likely that the evidence presented here is representative of the evidence base. There were 10 studies identified; these included one systematic review (97) and nine further original research studies (98–106) which employed a range of quantitative and qualitative methods, including surveys, interviews and focus groups (Supplementary Table S9).

### 6.1. Environmental barriers to cycling

The systematic review, which included 21 observational studies from five countries (14 from the USA, three from Australia, two from the UK, and one each from Canada and the Netherlands) focused on the effect of the environment on cycling levels (97). It reported that key environmental-level barriers to cycling included: perceived and objective traffic danger; distance from cycle paths; long trip distance and steep inclines, leading to; and high levels of effort.

Conversely, the presence of dedicated cycle routes or paths, separation of cycling from other traffic, high population density, short trip distance, and proximity of a cycle path or green space were positively associated with cycling (97). Similar environmental-level barriers to cycling were also reported by studies not included in the review. The main findings from these additional studies were that the main barriers to cycling were: low perceived safety (98–100, 102, 103); lack of cycling infrastructure (including secure cycle parking and shower facilities) (98–103); distance and perceived effort (100, 102, 106); and bad weather (98–100, 104, 106).

Related to safety, three studies further identified the perceived poor attitude of other road users as a barrier to cycling (98, 102, 103). They recommended education for other road users on safe interaction with cyclists, and the promotion of cycling as a mode of transport (not just a recreational activity) to help normalise it within a population.

One study reported that owning a car was negatively associated with using a bike as a mode of transport (101), and suggested making it less appealing to use a car (e.g., raising the cost of parking at work) may help to increase cycling amongst car owners.

One study used a modelling approach to predict that increases in numbers of people cycling would lead to greater political will to improve the cycling environment and a “safely in numbers” effect that would increase the perceived and actual safety of cycling, thereby creating a virtuous cycle for growth (103).

Finally, at an environmental level, the presence of dedicated cycle routes or paths, separation of cycling from other traffic, high population density, short trip distance, and proximity of a cycle path or green space appear to be positively associated with cycling (97).

### 6.2. Individual-level barriers to cycling

The studies that were not included in the review also reported on individual-level, barriers to cycling. These included: lack of skills and confidence (100, 101, 105); physical discomfort and impracticality of cycling (104–106); and cost (99), although cost-saving was also reported as a facilitator to cycling (98, 99).

In contrast to studies undertaken in areas with relatively weak infrastructure (98–103), in countries where cycling infrastructure was good (Netherlands, Belgium), individual-level factors were generally more important than environmental-level factors in predicting cycling behaviour (105, 106). While the relative importance of individual vs. environmental barriers will be context-specific, this evidence suggests that focusing solely on improving infrastructure may not be sufficient to maximise cycling participation.

Connell et al. conducted a full exploration of barriers for office workers for the development and optimisation of a workplace cycling intervention (107). They first nationally representative survey of UK adults, then undertook focus groups with bank employees to understand any context-specific barriers and ways in which these might be overcome. These activities led to identification of 10 individual-level, two social-level, and five organizational-level modifiable factors, which were mapped to candidate intervention components previously identified in a scoping review of cycling initiatives (5) This work led to the development of a tailored multi-component workplace cycling intervention designed to address as many barriers to cycling to enable a sustained uptake in cycling behaviour in bank employees.

There were a number of evidence gaps in the peer-reviewed literature, particularly on barriers to cycling in older adults and disabled populations. Most studies specifically examined cycling as a mode of transport (99–102, 105, 106) or explored barriers to transport and recreational cycling combined (97, 98, 103, 104). Information on perceived barriers to recreational cycling specifically (which are likely to differ from barriers to cycling for transport) was limited.

### 6.3. Summary of evidence on barriers to cycling

The main barriers to cycling operate on both environmental (lack of facilities and infrastructure, weather, and perceived effort due to long distances, hills) and individual [perceived lack of safety and lack of confidence, skills and time, and perceived inconvenience/ impracticality relative to alternatives (i.e., using the car)] levels.

Importantly, women may be more concerned about safety and lack confidence to a greater degree than men, and even where cycling infrastructure is good, individual barriers remain important.

In addition, the promotion of cycling as an everyday (useful, enjoyable and social) activity, rather than a sport will be needed to maximise uptake of cycling. A multi-faceted approach targeting different population groups in different contexts is therefore needed to reduce barriers to cycling and facilitate increased participation.

However, more research is needed to understand specific barriers to recreational cycling, and in different population groups (i.e., older people, specific disabilities, specific BAME groups). Table 1 summarises what we know and what is not yet know about barriers to cycling.

## 7. Potential solutions to increase the number of people cycling: what has worked, what hasn't worked, and what could be tried?

When implementing and evaluating potential solutions to increase the number of people cycling, it can be helpful to think about the “level” of the intervention. A useful and previously used (108) model is the social-ecological framework (109). The factors that can influence a behaviour (in this case cycling) and also the levels at which interventions and actions can be aimed at: the *individual* level (may include biological and psychological approaches); the *social* level (may include family, group, cultural and community approaches); interventions at the *physical environment* level (could include facilities and infrastructure); and the *policy* level (may include legislation, funding and national strategies and priorities).

### 7.1. The current evidence for the promotion of cycling

The evidence for the promotion of cycling draws from different study types; observational studies examining factors which influence cycling; experimental studies, empirical studies such as natural experiments, and evaluations of public health policies. In the past two decades there have been numerous attempts to synthesise this evidence. Table 3 provides an overview of the current evidence base for the promotion of cycling using key selected systematic reviews and public health guidance reports.

**Table 3:** An overview of key reviews on potential solutions and facilitators for promoting cycling.

Author	Level of intervention in the social-ecological model	Main findings
Kelly (5)	Individual Social	A scoping review of literature on individual, social, and organizational level interventions to improve cycling levels. The study creates a map to summarise the broad action types (described by Michie et al. 110) feasible for implementation within organization/group-based cycling promotion initiatives, to act as a critical tool for employers, communities, practitioners, and researchers in designing interventions to increase cycling.
Kärmeniemi (111)	Physical environment	A systematic review of before-and-after design studies to assess how impacting the built environment impacts on physical activity. New routes and bike lanes, traffic free routes, perceived access to destinations, bus-ways with parallel cycling paths, and reductions in perceived danger all predicted increases in cycling.
Winters (112)	Policy	Found evidence that policies related to active travel may operate at various levels of the socio-ecological framework, including society, cities, routes or individuals. The provision of convenient, safe and connected walking and cycling infrastructure is at the core of promoting active travel, but policies may work best when implemented in comprehensive packages.
Savan (113)	Social Individual	Based on a comprehensive literature review, key elements from the social psychology literature associated with successful cycling adoption initiatives were reported. Five key interlocking strategies were described: (1) strategic population segmentation; (2) identification and removal of barriers; (3) the use of commitment strategies, including the foot in the door and pledge techniques; (4) tactics to sustain behaviour change, including visual images, prompts, reminders, social cues and modelling, social norms, branding, feedback and incentives, and; (5) ongoing social support, through modelling, local hubs and community involvement.
Giles-Corti (114)	Physical environment Policy	This narrative review identified eight integrated interventions that are needed to create cities that promote (walking and) cycling. “Urban” and “Transport planning and design” policies were differentiated. Planning interventions included destination accessibility, employment distribution, and parking demand management. Urban design interventions included connective design, residential density, distance to public transport, land-use diversity, and neighbourhood desirability.
Fell and Kivinen (115)	Social Physical environment	This review reported that there is a widespread agreement in the literature that the most effective mechanisms for boosting cycling (and walking) comprise integrated and complementary packages of interventions. Infrastructure is generally regarded as necessary but not sufficient to boost cycling and walking; while behaviour change interventions in the absence of adequate enabling infrastructure are also judged unlikely to be effective. Effective interventions include; Personal travel planning, Cycle to Work days, Cycle-hire/bike-share schemes, Provision of dedicated cycling lanes (and bicycle parking) and Some school-based interventions. The best investment strategy may comprise a strategic, networked approach and is likely to comprise a mix of measures.
Stewart (116)	Social Physical environment	A systematic review of 12 studies which aimed to increase commuter cycling. Group level approaches: Three bike to work schemes had mixed, but generally positive effects. A self-help programme did not impact cycling, but a support programme that provided social support and bicycles had a large effect. A 2 month cycling training programme had no effect, while a 12 month programme did. Environmental approaches: A single infrastructure project (building a bridge) increased cycle commuting, while two city-wide infrastructure interventions had positive impacts. Two whole of city investment approaches had small positive effects that were considered difficult to detect.
Hunter (117)	Social Physical environment	A systematic review of 12 studies to promote physical activity in urban green space. An urban greenway trail designed to enhance connectivity of pedestrian infrastructure with nearby retail establishments and schools, showed increases in cycling. A promotion campaign of a newly constructed Rail Trail that included press ads, maps of trails, newspapers and local radio, brochures distributed to local organizations and schools, and a launch event showed that intervention group cyclists increased mean cycling time compared to control area cyclists, and mean bike counts on the trail increased after the trail launch.
Mayne (118)	Physical environment	A systematic review of natural or quasi-experiments to examine the effects of policy and built environment changes on obesity-related outcomes; 17 addressed physical activity. Bike lanes and off-street bike paths increased cycling in three out of four studies. Two studies found increased cycling after implementation of the London and Montreal bicycle share programmes.
Community Preventive Services Task Force (119)	Physical environment	A systematic review of 90 studies provided evidence for the effectiveness of cycling infrastructure including protected bicycle lanes, trails, traffic calming, intersection design, street lighting and landscaping.
Scheepers (120)	Individual Social Physical environment	This review reported interventions categorised as work-place, changes to urban design, population-wide, and bike sharing which were typically multi-component, including self-help materials, public awareness, social marketing campaigns, and workplace travel plans. Of 14 studies which reported effects on cycling, 10 reported increases in cycling. Increases in cycling were reported for an annual short term campaign, workplace travel plans (e.g. storage, subsidized bicycles, facilities), commuter cycling promotion, financial incentives, car-free city centres, town-wide initiatives, cycle proficiency classes, individualised marketing, smart bicycles, and bicycle sharing schemes. Negligible effects for neighbourhood trails, traffic tolls, national cycle networks, cycle paths.
Bird (121)	Individual	A systematic review investigated what individual level behaviour change techniques have been used to promote walking and cycling. Of 46 included studies, *n* = 16 reported combined walking and cycling findings (none were cycling only). While the findings were mixed, they generally supported the inclusion of self-monitoring and intention formation techniques in future walking and cycling intervention design.
National Institute for Health and Care Excellence (122)	Individual Social Physical Environment Policy	Guidance on how to increase (walking and) cycling. Policy and planning recommendations included ensuring high-level support from the health sector and ensuring all relevant policies and plans consider (walking and) cycling. Local action recommendations included developing programmes, community wide-programmes, and personalised travel planning. A focus on schools, workplaces and the NHS was also recommended. Other measures to tackle the wider influences on walking or cycling were recommended including measures to reduce road dangers and to reallocate road space to create a more supportive environment. The need to address health inequalities around (walking and) cycling was emphasised.
Fraser (97)	Physical environment	This review reported evidence from observational studies examining associations between cycling and the built environment. Positive associations were identified between cycling and dedicated cycle lanes and ‘safe routes to school programme’.
Yang (123)	Individual Social	A systematic review of actions to promote cycling. Promoting specifically cycling: an intensive individual intervention in obese women, high quality improvements to a cycle route network, and two multifaceted cycle promotion initiatives at town or city level were found to be associated with increases in cycling. An educational and promotional intervention for cycling to school did not impact school journeys but increased recreational cycling. A community based social marketing programme involving information provision, cycle training, free bike hire, and a Ride To Work Day campaign aimed to promote the use of existing cycle paths showed residents no overall increase cycling. Individualised marketing of “environmentally friendly” modes of transport: *n* = 16 interventions aimed to promote a shift from cars to environmentally friendly modes of transport (walking, cycling, and public transport) by providing information tailored to individual households’ interests and requirements and were associated with modest but generally consistent net increases in cycling trip frequency. Other interventions that targeted travel behaviour: A sustainable transport public awareness campaign involving leaflets, mass media, exhibitions and talks in schools in the context of improvements to local transport infrastructure saw modest increases in cycling. A car-share initiative saw small decreases in cycling. A financial incentive intervention for not using a car-parking space reported a very small increase in cycling.
Bauman (108)	Individual Social Physical environment Policy	An overview of interventions shown to be successful in Australia. These were shown to be; Mass marketing campaigns highlighting the benefits of cycling; Bicycle education programs to increase skills, confidence and safety; Behaviour change initiatives to market alternatives to car use; Cycling events to provide incentives for people to ride in a supportive environment particularly for novice riders; Urban planning; Improved bicycle infrastructure; and funding from all levels of government focused on increasing bicycle friendly design.
Ogilvie (124)	Individual Social Physical environment	A systematic review of studies which attempted to promote walking and cycling as an alternative to using a car. Results typically presented for combined walking and cycling however some evidence was found that targeted programmes led to behaviour change in motivated groups. There was inconclusive evidence for other intervention types such as publicity campaigns, engineering measures and financial incentives.

There are many potential solutions to increase cycling which exist across all four levels of the socio-ecological model and which may target different types of cycling. For example, interventions have attempted to increase commuter cycling at the level of the *individual* (123), longitudinal studies (observing people over time) provide evidence of how changes in the *physical environment* may lead to changes in leisure and/or transport cycling (111), and policies have been developed to promote active travel to impact at community, city and population levels (112).

Not all reviews in Table 3 report consistent findings in the expected direction for a particular intervention or action. This may be attributed to differences in: the aim of each review; the types of study included within each review; and the methodological quality of individual studies with a review.

It is also important to consider that some potential solutions may only have an impact on one type of cycling; as an example, the provision of new cycle paths in a city centre may lead to an increase in cycling for transport but have no effect on cycling for recreation, or vice versa. Additionally, when designing and evaluating interventions and policies to increase active transport cycling is often combined with walking; this can make it difficult to identify what actions are specifically targeted towards cycling and what the impact of the intervention is on cycling.

There are clearly some challenges to identifying the optimal solutions to promote cycling from the existing evidence base. However, acknowledging these challenges, it is possible to identify a range of promising and potential solutions from across the different levels of the socio-ecological model.

#### 7.1.1. The individual level

The evidence base is relatively small and inconsistent at this level. Several studies implemented walking and cycling actions in the form of *self-help materials* and *targeted behaviour change programmes* for active travel. These have been found to be effective in shifting trips being made by car to walking and cycling. However, few studies have robustly evaluated individual level intervention solutions for cycling in isolation; these actions are typically included as part of a multi-component or multi-behaviour strategy. Several studies evaluating *individualised travel planning* do report positive findings although it should be noted these typically contain several accompanying social and physical environment actions. There is mixed evidence for the impact of *financial incentives* on cycling although some studies do demonstrate short-term positive effects. There is little evidence that *education and awareness* campaigns by themselves are sufficient to prompt behaviour change. Such campaigns may be an important first step to changing attitudes around cycling, but additional accompanying actions to identify and remove barriers appear to be required.

#### 7.1.2. Social level solutions

At this level, the evidence base is still early in its development. Again, it is often difficult to identify actions that would be considered purely at the social level; there is often overlap with individual level actions or they contribute to a multi-component, community wide project also including changes to the physical environment. Currently, there is insufficient evidence to support the promotion of several potential solutions at this level by themselves in promoting cycling. Such actions include: workplace *transport planning,* one-off large-scale *cycling events* (e.g., a Ride to Work Day), conducting *workplace challenges*, and having *workplace cycling champions*.

Again there are instances where such programmes have been effective, and others where they have not. Whilst such solutions by themselves may not produce behaviour change, they may be useful for the purposes of reinforcing and reminding people about their behaviour, enhancing social support and contributing to the creation of a cycling culture within a community or organisation.

#### 7.1.3. Physical environment level solutions

The strongest and largest body of evidence exists at this level. Early reviews found inconsistent findings in associations between the physical environment and cycling, potentially due to a lack of studies and low methodological quality. More recent reviews reflect the increased research focus on the physical environment as an important determinant of physical activity behaviours such as cycling.

Observational evidence consistently demonstrates that several characteristics related to the design of the physical environment including the *connectivity of streets, distance and access to destinations, and perceived safety* are associated with levels of cycling. There is also emerging evidence that changes to these characteristics are associated with changes in cycling. Most recently, there has been an increase in more robust evaluations of cycling infrastructure projects—both in improvements to existing infrastructure and the creation of new infrastructure.

The evidence is becoming increasingly strong that introducing actions such as appropriate signage, local facilities (bicycle racks, showers etc), dedicated and high-quality cycle paths, comprehensive networks, and traffic calming measures leads to an increase in cycling. It should be noted that such infrastructure improvements may work best when combined with actions at other levels of the socio-ecological model. There is also a substantial body of evidence emerging, demonstrating the consistently positive effects of bicycle hire and sharing schemes within organisations and at wider community and city levels. However, it is important to recognise, as Fell and Kivenen state, that “Infrastructure is generally regarded as necessary but not sufficient to boost cycling” (115).

#### 7.1.4. Policy level solutions

The evidence on the impact of interventions at the policy level is the least developed. Much of the evidence appraised by the key reviews in Table 3, proposes potential policies that could be created and implemented based around interventions at other levels of the social-ecological model (i.e., utilising policy-related evidence). For example, using the evidence base around improvements to infrastructure and the physical environment to suggest policies should be generated to improve cycle routes. Similarly, several reviews recommend an increase in government investment in cycling related projects to support attempts to increase cycling but without a formal evaluation of any previous funding strategies. However, there is some mixed evidence which suggests that introducing policy and legislation to lower vehicle speed limits (i.e., introduction of 20 mph zones and limits) may result in an increase in cycling.

### 7.2. Summary of solutions to increase the number of people cycling

Cycling is a complex, multi-faceted behaviour, with barriers and facilitators operating at multiple levels for different individuals and sub-groups of the population. Accordingly, in order to address these barriers, multiple solutions will be required. What is clearly evident from this overview of the evidence base is that no single intervention, programme or policy will be sufficient to produce long-lasting, population-wide increases in cycling.

Research has demonstrated that it is essential to target both the “*place*” through improving cycling infrastructure at the physical environmental level, and also the “*person*” through individual and social level support. Whilst intervening at the level of the physical environment and improving the infrastructure for cycling is possible and undoubtedly necessary, doing so in isolation is unlikely to be sufficient to prompt behaviour change for many individuals. Thus, what will be needed to best promote cycling has been described as “*a coordinated package of complementary infrastructure measures, programs and policies” (*77). It will also require time following introduction of such packages in order for cycling to become normalised and imbedded in a workplace or in society in general.

However, large-scale infrastructure and national policy changes require substantial financial and political investment and are simply not feasible for several organisations such as schools, business, charities and workplaces who may wish to promote cycling. Therefore, it is imperative that there is further development and a rigorous examination of the types of interventions and programmes that these stakeholders can implement—namely those at the individual and social levels. Critically, this is where the evidence base is weakest and it has been acknowledged that there is “*a paucity of studies that investigate and rigorously evaluate the independent effects of behaviour-based cycling promotion initiatives*” (113).

Table 4 provides a summary of what we know and what is not yet know about how best to promote cycling. The existing evidence base for the promotion of cycling is not complete, and this limits our ability to affect the sort of change that would impact population health. In addition to the limitations of the current evidence base noted above, there are two important reasons why the existing evidence base does not give the full picture.

**Table 4:** What is known and what is not yet know about how best to promote cycling.

Solutions to promote cycling		
What is known	What is not yet known	What is needed to fill evidence gap
• Numerous systematic reviews exist which synthesise the evidence from observational and experimental studies and which highlight that there are many potential solutions to promote cycling. These exist across all four levels of the socio-ecological model (individual, social, physical environment and policy). The most likely way to promote cycling is through an integrated package of complementary actions targeting both individuals and their social and physical environments. • The evidence base appears largest and strongest at the level of the physical environment, where emerging evidence from robustly evaluated natural experiments supports the creation of new cycle paths, routes and networks which requires considerable financial investment and political support from local and national governments.	• The current evidence base is built primarily from systematic reviews of studies found in academic journals. Hence, publication bias and a focus on programmes considered easiest to evaluate has likely led to an incomplete picture of how best to promote cycling. Many potential solutions, delivered outside of an academic setting, have not been studied or evaluated. Additionally, there are several novel potential solutions emerging for which the effectiveness is not yet understood. • Where the evidence base is less abundant and consistent is at the individual and social levels of the socioecological model. Some evidence exists for targeted behaviour change programmes but the evidence is inconclusive for interventions such as financial incentives, counselling and education and awareness raising. There have been few rigorous evaluations of the independent effects of such efforts to promote cycling yet these are the types of programmes that organisations such as schools and workplaces can implement.	• There is a need to build a comprehensive picture of all potential solutions to increase cycling, at the levels appropriate for delivery by organisations such as workplaces. Whilst such solutions may be considered smaller in scale than large infrastructure projects, they could still be delivered nationwide across workplaces, communities, and schools. They may also encourage the use of existing infrastructure. They are therefore undoubtedly an essential component of the integrated package of measures that will be required to increase population levels of cycling. • A comprehensive picture is required to highlight the most promising, novel and feasible solutions to take forward for subsequent pilot and preference testing. It will be necessary to examine these solutions for their potential in addressing the barriers to cycling faced by different individuals and population sub-groups.

First, since the evidence base is comprised of what has been studied and evaluated, typically in an academic setting. Therefore, it will always tend to be influenced by what is quick, convenient, and cheap to study and evaluate. It is likely to be further influenced by what is of interest to academic journals and research funders (this is known as publication bias). In short, not all things get evaluated, and not all things that get evaluated make it into journals and reports. The outcome is that the “accepted” evidence base can give a very limited picture of the full scope of approaches.

Second, there are some novel and fast changing approaches, for which the effectiveness is not yet fully understood. These include potential strategies such as; the introduction of e-Bikes (electric assisted bicycles) (125), the use of evolving technologies such as Smart Phone applications (126), and Intelligent Transport using the Internet of Things (127).

There is a need to understand how to increase cycling through interventions that can be delivered effectively, cost-effectively and at scale to benefit population health. For the reasons given above, it is important to look beyond what is already evaluated and deemed effective. It is clear that a critical next step is to build the evidence base in terms of possible approaches to promote cycling. An efficient way to do this would be to undertake a scoping review of all existing approaches utilising traditional electronic record searching, and importantly the databases of key cycling promotion organisations, and expert stakeholder consultation. This would generate a more comprehensive evidence base, identifying interventions which have been rigorously evaluated and published and also interventions which have not been evaluated but may provide alternative and potentially novel solutions to promote cycling. This would facilitate mapping of the actions and functions of the different intervention approaches (the active ingredients) as well as specified intervention characteristics including; duration (how long the intervention lasts for), scale (at level of socio-ecological model), setting (e.g., workplace, community, school, etc.), and target population (e.g., adults).

**Figure 3:**
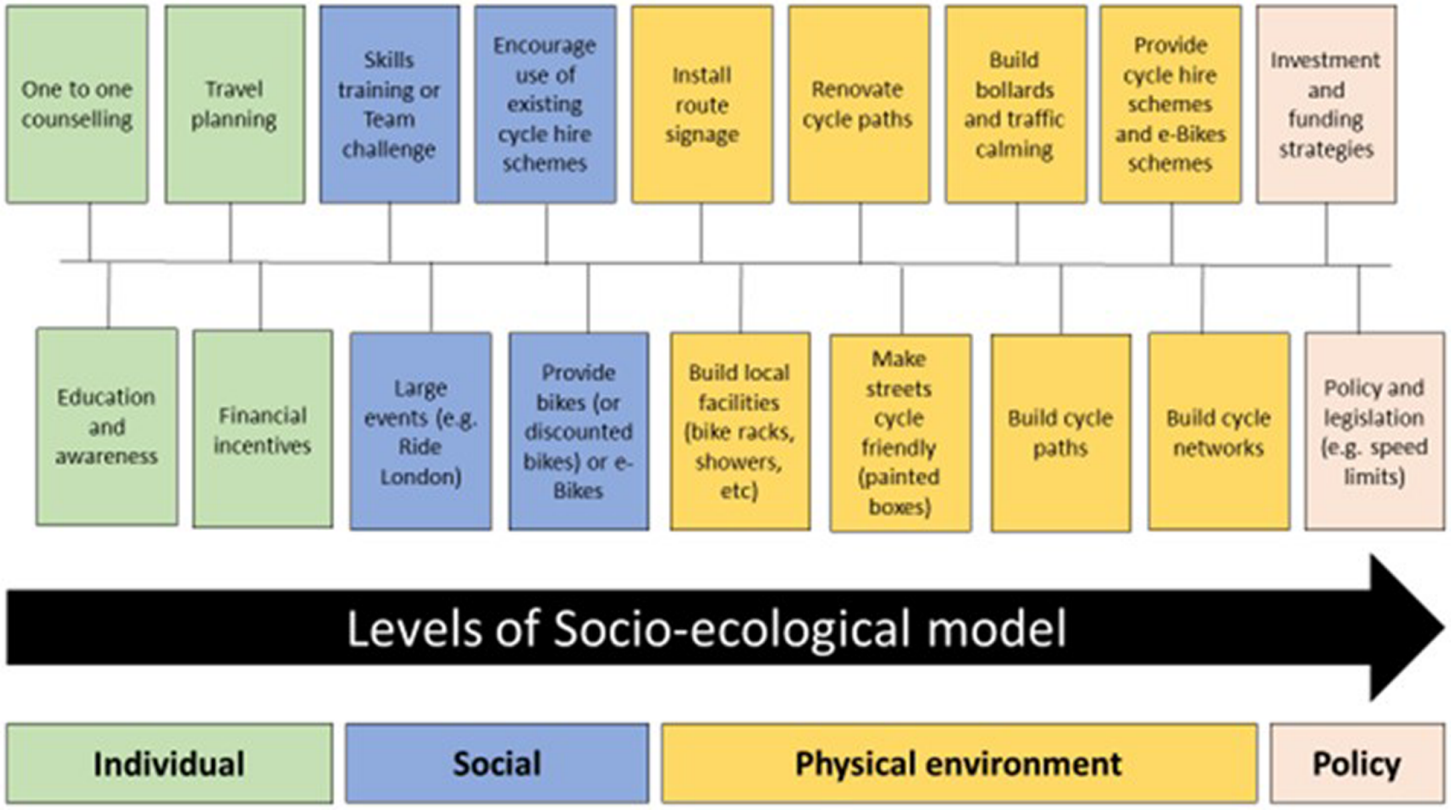
Interventions and actions to promote cycling mapped on the socio-ecological model.

A conceptual version of this is presented in Figure 3, which maps intervention actions against the socio-ecological model. It is apparent from this figure that moving up the socio-ecological model into Physical Environment (yellow) and Policy (pink), the implementation of the actions requires greater resource and influence. As stated, actions at these levels are likely to be necessary, but not sufficient, to induce substantial shifts in cycling participation at the population level, and actions at the Individual (green) and Social (blue) levels are also likely to be needed. Thus, a map of potential interventions across the socio-ecological model will enable identification of actions which are feasible for implementation by organisations, workplaces, schools, and local stakeholders and can complement interventions at the level of policy and the macro-physical environment and provide the base for preference testing and ultimately intervention piloting, on the route to creating an evidence based tool-kit for promoting cycling.

### 7.3. Case study: the cycle nation project

In recognition of the need to address barriers at different levels of the socio-ecologial model, the Cycle Nation Project (CNP) aimed to develop, test the feasibility of, and optimise a multi-component workplace-based intervention to increase cycling among office staff at a multinational bank (107). To ensure that the most appropriate strategies were utilised in the intervention, focus groups were conducted with bank employees to understand any context-specific barriers and ways in which these might be overcome. This led to identification of 10 individual-level, two social-level, and five organizational-level modifiable factors, which were mapped to candidate intervention components previously identified in the Kelly et al. scoping review of cycling initiatives (5). The resultant pilot intervention included 32 core components across six intervention functions (education, persuasion, incentivisation, training, environmental restructuring, enablement). Participants received a loan bike for 12-weeks (or their own bike serviced), and a 9-week cycle training course (condensed to 6 weeks for those already confident in basic cycling skills), including interactive information sharing activities, behaviour change techniques (e.g., weekly goal setting), bike maintenance training, practical off-road cycling skill games and on-road group rides. To address the sustainability of the intervention, sessions were delivered by trained bank staff members who were experienced cyclists.

The CNP pilot intervention was delivered across three sites with 68 participants. It was completed in two sites (the third site was stopped due to COVID-19) and was feasible and acceptable to both women and men and across different ethnicities. In addition, the CNP intervention was successful (at least in the short term) in increasing cycling by 3 rides/week on average, and improving perceptions of safety, vitality, confidence, and motivation to cycle (107). This case study affirms how targeted intervention strategies can be applied to specific populations to successfully improve cycling behaviours. For successful large-scale rollout, there is need for development and evaluation of interventions, like the CNP, which use multi-component strategies.

## 8. Summary

Given the potential positive impact sustained uptake in cycling can have on public health, wellbeing, the economy, and the environment, it is important to invest in effective solutions to improve cycling behaviour. Although still in its infancy, research insights into behaviour change strategies show potential to foster increased cycling levels when applied to target populations. From reviewing the available literature, we observed that programme design for interventions to increase cycling may be most effective when a multi-component approach is utilised. Therefore, there is further need to develop and evaluate multi-component cycling interventions for successful large-scale application to maximise cycling uptake.
