# Supplementary material for: Benefits, risks, barriers, and facilitators to cycling: a narrative review
Source: Front Sports Act Living. 2023 Sep 19;5:1168357. doi: 10.3389/fspor.2023.1168357 (PMC10546027; doi:10.3389/fspor.2023.1168357)
Supplement: Supplementary file 1 [file Datasheet1.docx]

**Supplementary Table 1. Key features of the studies included in the Kelly et al (2014) systematic review and meta-analysis. Modified from** [3]

| **Author, Date** | **Country** | **Number of participants** | **Age range** | **Cycling domains** | **Length of follow-up (years)** | **Statistical adjustments performed** | **Key findings** |
| --- | --- | --- | --- | --- | --- | --- | --- |
| Sahlqvist (2013) | UK | 22450 adults | 40-79 years | Self-reported total cycling | 15.3 years | Age, sex, smoking, education, social class, other physical activity, family history of cardiovascular disease or cancer | Compared with undertaking no cycling, cycling between 1 and 59 minutes per week, and cycling at least 60 minutes per week were associated with lower risk of all-cause mortality of 4% and 9%, respectively, with the latter reduction being statistically significant. |
| Johnsen (2013) | Denmark | 26576 men  29129 women | 50-64 years | Self-reported cycling | 15.6 years | Age, sex, smoking, education, other physical activity, body mass index, history of cardiovascular disease or stroke, cholesterol, blood pressure, follow-up time | Compared with undertaking no cycling, participation in any cycling was associated with a 21% lower risk of all-cause mortality in women and a 9% lower risk of CVD mortality in men. |
| Schnohr (2012) | Denmark | 5106 adults reporting some cycling | 21-90 years | Self-reported duration and intensity of cycling | 18 years | Age, sex, smoking, education, income, other sports activities, blood pressure, diabetes, body mass index, blood lipids, alcohol intake | Participants were grouped according to duration of cycling (<0.5, 0.5-1, >1 hour per day) and cycling intensity (self-reported as slow, average, or fast). Faster pace of cycling was associated with lower risk of all-cause mortality in a dose-dependent manner. Duration of daily cycling was only weakly associated with risk of all-cause mortality. |
| Andersen (2011) | Denmark | 8466 men  6510 women | 20-93 years | Self-reported cycling commuting | 16 years | Age, sex, smoking, education, other physical activity, body mass index, cholesterol, blood pressure | Compared with undertaking no cycling, undertaking up to 3 hours/week, 3-7 hours/week, and over 7 hours/week of cycling was associated with 22%, 24% and 30% lower risk of all-cause mortality, respectively. |
| Besson (2008) | UK | 14903 adults | 45-79 years | Self-reported cycling for transportation | 7 years | Age, sex, smoking, social class, other physical activity, history of cancer, cardiovascular disease, stroke or diabetes, alcohol intake | Compared with no cycling, cycling for up to 30 minutes per week for transportation was associated with a numerically 2% higher risk of all-cause mortality, and cycling more than 30 minutes per week for transportation was associated with a numerically 1% lower risk of all-cause mortality, but these differences were not statistically significant. |
| Matthews (2007) | China | 67143 women | 40-70 years | Self-reported cycling to work and cycling for other reasons | 5.7 years | Age, smoking, education, income, other physical activity, chronic medical conditions, alcohol intake, number of pregnancies, oral contraceptive use, menopausal status | Compared with no cycling, cycling for up to 30 minutes per day was associated with a 21% lower risk of all-cause mortality, and cycling more than 30 minutes per day was associated with a numerically 34% lower risk of all-cause mortality. |
| Andersen (2000) | Denmark | 17265 men  13375 women | 20-93 years | Self-reported cycling to work | 13.8 years (women)  15.1 years (men) | Age, sex, smoking, education, leisure-time physical activity, body mass index, blood lipids, blood pressure | Compared with not cycling to work, cycling to work (on average 3 hours/week) was associated with 30% lower risk of all-cause mortality. |

**Supplementary Table 2 Major prospective cohort epidemiological studies, published since the Kelly et al (2014) systematic review and meta-analysis** [3]**, examining the association between cycling participation and all-cause mortality.**

| **Author, Date** | **Country** | **Number of participants** | **Age range** | **Cycling domains** | **Length of follow-up (years)** | **Statistical adjustments performed** | **Key findings** |
| --- | --- | --- | --- | --- | --- | --- | --- |
| Koolhaas (2018) | Netherlands | 7225 adults | 55 years and older (mean age 70 years) | Self-reported cycling | 13.1 years | Age, sex, smoking, education, other physical activity, alcohol consumption, diet quality, marital status, current diseases (cardiovascular disease, diabetes, cancer, chronic obstructive pulmonary disease) | Compared to undertaking no cycling, medium levels of cycling (median of 13 minutes per day) was associated with a 28% lower risk of mortality, and high levels of cycling (median 51 minutes per day) was associated with 35% lower risk of mortality. |
| Celis-Morales (2017) | UK | 263540 adults | 40-69 years | Self-reported cycling to work | 5.0 years | Age, sex, ethnicity, smoking, socio-economic deprivation, other physical activity, body mass index, dietary intake, long-standing illness, diabetes, hypertension, depression. Participants with cardiovascular disease or cancer at baseline were excluded from the analysis. | Compared with commuting using non-active forms of transport, cycling to work was associated with a 41% lower risk of mortality. Mixed-mode commuting, involving cycling part of the way to work with non-active travel for the remainder, was associated with a 24% lower risk of mortality. Both longer (more than 30 miles per week) and shorter (less than 30 miles per week) cycle commutes were associated with lower risk of mortality than a non-active commute (by 40% and 32%, respectively) |
| Oja (2016) | UK | 80306 adults | Mean age 52 years | Self-reported cycling for any purpose | 9.2 years | Age, sex, smoking, alcohol intake, education, other physical activity, body mass index, longstanding illness, cardiovascular disease, psychological distress | Compared to undertaking no cycling, participation in any cycling was associated with a 15% lower risk of mortality. There was no clear effect of self-reported cycling intensity or weekly cycling duration on this association. |
| Andersen (2015) | Denmark | 52061 adults | 50-65 years | Self-reported leisure time and transport-related cycling | 13 years | Age, smoking, sex, education, mean income in residential area, year of measurement, other physical activity, smoking, environmental tobacco smoke, diet, alcohol intake, marital status, occupational risk of pollution exposure, exposure to NO_2_ at residential address | Compared to undertaking no cycling, participation in any cycling was associated with a 17% lower risk of mortality. This was association was not modified by exposure to traffic-related air pollution. |

**Supplementary Table 3 Major prospective cohort epidemiological studies examining the association between cycling participation and cardiovascular disease incidence and mortality.**

| **Author, Date** | **Country** | **Number of participants** | **Age range** | **Cycling domains** | **Length of follow-up (years)** | **Statistical adjustments performed** | **Key findings** |
| --- | --- | --- | --- | --- | --- | --- | --- |
| Kubesch (2018) | Denmark | 51868 adults | 50-64 years | Self-reported leisure time and utilitarian cycling | 17.7 years | Age, sex, smoking, education, other physical activity, diet, alcohol consumption, environmental tobacco smoke, marital status, hormone replacement therapy use. | Compared with no cycling, participation in cycling was associated with a 9% lower risk of a first myocardial infarction (heart attack) and a 20% lower risk of a recurrent myocardial infarction. Amongst those who cycled, cycling from 0.5 to 4 hours per week, and cycling more than 4 hours per week were associated with 7% lower and 14% lower risk, respectively, of a first myocardial infarction compared with those cycling less than 0.5 hours per week. Participation in 0.5 to 4 hours of cycling per week was associated with a 31% lower risk of a recurrent myocardial infarction than cycling less than 0.5 hours per week, but there was no statistically significant difference in risk of a myocardial infarction between those cycling less than 0.5 hours and more than 4 hours per week. |
| Celis-Morales (2017) | UK | 263540 adults | 40-69 years | Self-reported cycling to work | 2.1 years | Age, sex, ethnicity, smoking, socio-economic deprivation, other physical activity, body mass index, dietary intake, long-standing illness, diabetes, hypertension, cancer, depression. Participants with CVD at baseline excluded from analysis. | Compared with commuting using non-active forms of transport, cycling to work was associated with a 46% lower risk of CVD incidence and 52% lower risk of CVD mortality. Mixed-mode commuting, involving cycling part of the way to work with non-active travel for the remainder, was associated with a numerically 18% lower risk of CVD incidence and a numerically 8% lower risk of CVD mortality, but these associations were not statistically significant. When cycling commuting was stratified by distance (more than 30 miles per week vs less than 30 miles per week) statistically significant reductions in CVD incidence and CVD mortality only observed with the longer weekly commuting distance. |
| Oja (2016) | UK | 75014 adults | Mean age 52 years | Self-reported cycling for any purpose | 9.2 years | Age, sex, smoking, alcohol intake, education, other physical activity, body mass index, longstanding illness, psychological distress. Participants with CVD at baseline excluded from analysis. | Compared with undertaking no cycling, participation in any cycling was associated with a numerically 7% lower risk of CVD mortality, but this was not statistically significant. |
| Blond (2016) | Denmark | 53723 adults | 50-65 years | Self-reported leisure time and commuter cycling assessed at baseline and again approximately 5 years later | 20 years | Age, sex, education, smoking, other physical activity, diet, alcohol intake, body mass index, diabetes, medications for high cholesterol or high blood pressure.  Participants with CHD, stroke or cancer at baseline excluded from analysis | Compared with undertaking no cycling, participation for up to 1 h/week, 1-2.5 h/week, and over 2.5 h/week of overall cycling at baseline was associated with 13%, 8% and 14% lower risk of CHD incidence, respectively. Leisure time cycling for up to 1 h/week, 1-2.5 h/week, and over 2.5 h/week of overall cycling at baseline was associated with 16%, 20% and 20% lower risk of CHD incidence, respectively Commuting cycling for up to 1.5 h/week and more than 1.5 h/week was associated with 19% and 6% lower CHD risk, respectively, but these were not statistically significant.  Compared with no cycling at either time-point, initiatinginitiatingi cycling at the second time-point after not cycling at baseline was associated with 24% lower CHD risk, and cycling at both time-points was associated with 20% lower CND risk. Cycling only at baseline was associated with 12% lower CHD risk, which was not statistically significant. |
| Andersen (2015) | Denmark | 52061 adults | 50-65 years | Self-reported leisure time and transport-related cycling | 13 years | Age, smoking, sex, education, mean income in residential area, year of measurement, other physical activity, smoking, environmental tobacco smoke, diet, alcohol intake, marital status, occupational risk of pollution exposure, exposure to NO_2_ at residential address | Compared to undertaking no cycling, participation in any cycling was associated with a 22% lower risk of CVD mortality. |
| Johnsen (2013) | Denmark | 26576 men  29129 women | 50-64 years | Self-reported cycling | 15.6 years | Age, sex, smoking, education, other physical activity, body mass index, history of cardiovascular disease or stroke, cholesterol, blood pressure, follow-up time | Compared with undertaking no cycling, participation in any cycling was associated with a 24% lower risk of CVD mortality in women and a 14% lower risk of CVD mortality in men. |
| Sahlqvist (2013) | UK | 22450 adults | 40-79 years | Self-reported total cycling | 15.3 years | Age, sex, smoking, education, social class, other physical activity, family history of cardiovascular disease or cancer | Compared with undertaking no cycling, cycling between 1 and 59 minutes per week, and cycling at least 60 minutes per week were associated with numerically lower risk of CVD mortality of 17% and 13%, respectively, but these reductions were not statistically significant. |
| Schnohr (2012) | Denmark | 5106 adults reporting some cycling | 21-90 years | Self-reported duration and intensity of cycling | 18 years | Age, sex, smoking, education, income, other sports activities, blood pressure, diabetes, body mass index, blood lipids, alcohol intake | Participants were grouped according to duration of cycling (<0.5, 0.5-1, >1 hour per day) and cycling intensity (self-reported as slow, average, or fast). Faster pace of cycling was associated with lower risk of death from coronary heart disease in a dose-dependent manner. Duration of daily cycling was not associated with risk of coronary heart disease death. |
| Hoevenaar-Blom (2011) | Netherlands | 7451 men  8991 women | 20-65 years | Self-reported cycling | 9.8 years | Age, sex, smoking, education level, other physical activity, alcohol consumption | Compared with no cycling, participation in cycling was associated with an 18% lower risk of CVD incidence. Cycling for up to 3.5 hours per week or more than 3.5 hours per week were both associated with 18% lower risk than no cycling participation. |
| Besson (2008) | UK | 14903 adults | 45-79 years | Self-reported cycling for transportation | 7 years | Age, sex, smoking, social class, other physical activity, history of cancer, cardiovascular disease, stroke or diabetes, alcohol intake | Compared with no cycling, cycling for up to 30 minutes per week for transportation was associated with a numerically 19% lower risk of CVD mortality, and cycling more than 30 minutes per week for transportation was associated with a numerically 28% lower risk of CVD mortality, but these reductions were not statistically significant. |
| Matthews (2007) | China | 67143 women | 40-70 years | Self-reported cycling to work and cycling for other reasons | 5.7 years | Age, smoking, education, income, other physical activity, chronic medical conditions, alcohol intake, number of pregnancies, oral contraceptive use, menopausal status | Compared with no cycling, cycling for up to 30 minutes per day was associated with a numerically 25% lower risk of CVD mortality, and cycling more than 30 minutes per day was associated with a numerically 37% lower risk of CVD mortality, but these reductions were not statistically significant. |
| Tanasescu (2002) | USA | 44452 men | 40-75 years | Self-reported cycling | 10.7 years | Age, smoking, alcohol intake, other physical activity, body mass index, family history of myocardial infarction, diet, use of vitamins, diabetes, hypertension, hypercholesterolaemia | No significant association of participation in cycling on risk of coronary heart disease. |

**Supplementary Table 4. Major prospective cohort epidemiological studies examining the association between cycling participation and cancer incidence and mortality.**

| **Author, Date** | **Country** | **Number of participants** | **Age range** | **Cycling domains** | **Length of follow-up (years)** | **Statistical adjustments performed** | **Key findings** |
| --- | --- | --- | --- | --- | --- | --- | --- |
| Celis-Morales (2017) | UK | 263540 adults | 40-69 years | Self-reported cycling to work | 2.1 years | Age, sex, ethnicity, smoking, socio-economic deprivation, other physical activity, body mass index, dietary intake, long-standing illness, diabetes, hypertension, CVD, depression. Participants with cancer at baseline excluded from analysis. | Compared with commuting using non-active forms of transport, cycling to work was associated with a 45% lower risk of cancer incidence and 40% lower risk of cancer mortality. Mixed-mode commuting, involving cycling part of the way to work with non-active travel for the remainder, was associated with a 32% lower risk of cancer incidence and a 36% lower risk of cancer mortality. When cycling commuting was stratified by distance (more than 30 miles per week vs less than 30 miles per week) statistically significant reductions in cancer incidence and cancer mortality only observed with the longer weekly commuting distance. |
| Andersen (2015) | Denmark | 52061 adults | 50-65 years | Self-reported leisure time and transport-related cycling | 13 years | Age, smoking, sex, education, mean income in residential area, year of measurement, other physical activity, smoking, environmental tobacco smoke, diet, alcohol intake, marital status, occupational risk of pollution exposure, exposure to NO_2_ at residential address | Compared to undertaking no cycling, participation in any cycling was associated with a 7% lower risk of cancer mortality, but this was not statistically significant. |
| Johnsen (2013) | Denmark | 26576 men  29129 women | 50-64 years | Self-reported cycling | 15.6 years | Age, sex, smoking, education, other physical activity, body mass index, history of cardiovascular disease or stroke, cholesterol, blood pressure, follow-up time | Compared with undertaking no cycling, participation in any cycling was associated with a 10% lower risk of cancer mortality in women and a 1% lower risk of cancer mortality in men, but these reductions were not statistically significant. |
| Sahlqvist (2013) | UK | 22450 adults | 40-79 years | Self-reported total cycling | 15.3 years | Age, sex, smoking, education, social class, other physical activity, family history of cardiovascular disease or cancer | Compared with undertaking no cycling, cycling between 1 and 59 minutes per week, and cycling at least 60 minutes per week were associated with numerically lower risk of cancer mortality of 1% and 7%, respectively, but these reductions were not statistically significant. |
| Johnsen (2009) | 8 European countries (Denmark, Germany, Greece, Italy, Netherlands, Spain, Sweden, UK) | 127923 men | 20-97 years | Self-reported cycling to work, for shopping and in leisure time | 8.5 years | Age, education, other physical activity, height, weight, marital status. Participants with cancer at baseline were excluded. Smoking, diet, and alcohol intake, did not alter the associations or were not associated with prostate cancer risk, so were not included in statistical model. | Compared with undertaking no cycling, participation in cycling was associated a 3% lower risk of prostate cancer incidence, but this was not statistically significant. |
| Besson (2008) | UK | 14903 adults | 45-79 years | Self-reported cycling for transportation | 7 years | Age, sex, smoking, social class, other physical activity, history of cancer, cardiovascular disease, stroke or diabetes, alcohol intake | Cycling was not related to cancer mortality. |
| Matthews (2007) | China | 67143 women | 40-70 years | Self-reported cycling to work and cycling for other reasons | 5.7 years | Age, smoking, education, income, other physical activity, chronic medical conditions, alcohol intake, number of pregnancies, oral contraceptive use, menopausal status | Compared with no cycling, cycling for up to 30 minutes per day was associated with an 18% lower risk of cancer mortality, and cycling more than 30 minutes per day was associated with a numerically 45% lower risk of cancer mortality. The trend for lower cancer risk with increasing levels of cycling was statistically significant. |
| Johnsen (2006) | Denmark | 26122 men  28356 women | 50-64 years | Self-reported leisure time cycling | 7.6 years | Age, sex, smoking, education, occupational physical activity, body mass index, diet, alcohol intake, use of non-steroidal anti-inflammatory drugs, use of hormone replacement therapy. All participants were free from cancer at baseline. | Compared with no cycling, participation in cycling was associated with an 11% lower risk of colon cancer incidence in women and 8% lower risk in men, but these were not statistically significant. |
| Steindorf (2006) | 10 European countries (Denmark, France, Germany, Greece, Italy, Netherlands, Norway, Spain, Sweden, UK) | 416277 adults | most participants 35-70 years | Self-reported recreational cycling | 6.3 years | Age, sex, smoking, education, height, weight, diet, alcohol intake, occupational exposure to lung carcinogens, measurement centre. All participants were free from cancer (other than non-melanoma skin cancer) at baseline. | Compared with no cycling, participation in cycling was associated with lower risk of lung cancer in women (by 27%) but not in men. |

**Supplementary Table 5. Major prospective cohort epidemiological studies examining the association between cycling participation and type 2 diabetes incidence.**

| **Author, Date** | **Country** | **Number of participants** | **Age range** | **Cycling domains** | **Length of follow-up (years)** | **Statistical adjustments performed** | **Key findings** |
| --- | --- | --- | --- | --- | --- | --- | --- |
| Rasmussen (2016) | Denmark | 24623 men  27890 women | 50-64 years | Self-reported total cycling, recreational cycling and commuter cycling assessed at baseline and again approximately 5 years later. | 14.2 years | Age, sex, smoking, education, other physical activity, diet, alcohol intake, family history of diabetes. All participants were free from type 2 diabetes and other chronic diseases at baseline. Additional analyses also adjusted for body mass index and waist circumference. | Compared with no cycling, total cycling for 1-60, 61-150, 151-300 and > 300 min/week was associated with 13%, 17%, 20%, and 20% lower risk of developing type 2 diabetes. These associations were attenuated by adjustment for waist circumference (to 3%, 7%, 8% and 10% lower risk, respectively for increasing levels of cycling) or body mass index (to 4%, 10%, 11%, and 15% lower risk) after adjustment for waist circumference, indicating that some of the benefit of cycling was mediated through lower adiposity.  Cycling in ‘summer and winter’ was associated with lower diabetes risk (12% lower risk than no cycling) than cycling in ‘summer or winter’ (4% lower risk).  Compared with undertaking no cycling at both assessments, cycling at the first, but not second time-point was associated with 12% lower risk of type 2 diabetes; initiation of cycling at the second time-point was associated with 20% lower risk; and cycling at both time-points was associated with 29% lower risk.  Commuting cycling for 1-60, 61-150 and > 150 min/week was associated with 28%, 17% and 30% lower risk of type 2 diabetes compared with no commuting cycling. |
| Villegas (2006) | China | 70658 women | 40-70 years | Self-reported cycling | 4.6 years | Age, smoking, income, education, occupation, alcohol, pre-existing chronic disease (coronary heart disease, stroke, cancer), hypertension. All participants were free from type 2 diabetes at baseline. | Compared with no cycling, participation in cycling was associated with a 19% lower risk of type 2 diabetes.  In a sensitivity analysis in which all participants with chronic disease at baseline were excluded, participation in cycling was associated with a 14% lower risk of type 2 diabetes, which was no longer statistically significant. |
| Hu (1999) | USA | 70102 women | 40-65 years | Self-reported cycling | 8 years | Age, smoking, other physical activity, alcohol intake, menopausal status and post-menopausal hormone use, parental history of diabetes, cholesterol, blood pressure.  All participants were free from type 2 diabetes, cardiovascular disease and cancer (except non-melanoma skin cancer) at baseline. | Compared with no cycling, participation in cycling was associated with a 4% lower risk of type 2 diabetes, but this was not statistically significant. |

**Supplementary Table 6. Major prospective cohort epidemiological studies examining the association between cycling participation and indices of adiposity.**

| **Author, Date** | **Country** | **Number of participants** | **Age range** | **Cycling domains** | **Length of follow-up (years)** | **Statistical adjustments performed** | **Key findings** |
| --- | --- | --- | --- | --- | --- | --- | --- |
| Rasmussen (2018) | Denmark | 9014 men  8661 women | 50-64 years | Self-reported total cycling, recreational cycling and commuter cycling assessed at baseline and again approximately 5 years later. | 5.4 years | Age, sex, smoking, education, other physical activity, diet, length of follow-up, and baseline weight, BMI or waist circumference | In those without abdominal obesity at baseline (waist <102 cm in men and <88 cm in women), initiating cycling (i.e. cycling at the second by not first assessment) was associated with 15% lower risk of developing abdominal obesity and continuing cycling (i.e. cycling at both assessments) was associated with 18% lower odds of developing abdominal obesity.  In those who were not obese at baseline (BMI < 30 kg.m^-2^) continuing cycling was associated with 26% lower odds of developing obesity. Initiation of cycling did not significantly lower odds of developing obesity.  Continuing cycling was associated with a 0.53 cm smaller change in waist circumference over the follow-up period, but was not significantly associated with change in body weight. |
| Grøntved (2016) | Sweden | 23732 adults | 40-60 years | Self-reported commuter cycling by season at baseline and at 10-year follow-up | 10 years | Age, sex, smoking, education, other physical activity, dietary intake, follow-up time, baseline BMI or waist circumference | Cycling to work at baseline was associated with 15% lower odds of developing obesity, in those who were not obese at baseline. Only those who cycled in all four seasons has lower obesity risk.  Compared with those reporting passive commuting at both baseline and follow-up, initiating cycle commuting (i.e. passive commuting at baseline, cycle commuting at follow-up) was associated 36% lower odds of developing obesity. Continuing cycling was associated with 57% lower odds of obesity compared to switching to passive commuting.  Those who cycled to work at baseline had a 0.16 kg.m^-2^ smaller gain in BMI over the follow-up period compared to passive commuters. |
| Mytton (2016) | UK | 1451 adults |  | Self-reported cycling to work, assessed twice a year apart | 1 year | Age, sex, education, other physical activity, distance to work, physical wellbeing, year of study. Analyses conducted with and without adjustment for baseline BMI. | Compared with not cycling to work, maintaining cycling to work at both time-points was associated with 1.14 kg.m^-2^ lower BMI at follow-up in analyses not adjusted for baseline BMI but this was attenuated to 0.12 kg.m^-2^ lower BMI at follow-up, after baseline BMI adjustment, which was no longer statistically significant.  There were no significant associations between change in weekly cycle commute time and change in BMI. |
| Lusk (2010) | USA | 18414 women | 25-42 years | Self-reported cycling at baseline and 16-year follow-up | 16 years | Smoking, diet, alcohol intake, oral contraceptive use, parity, anti-depressant use | Women who reported no cycling at baseline and had initiated cycling by follow-up has less weight gain than those who reported no cycling at either time-point, in a dose-dependent manner, with those reporting up to 5 mins/day cycling at follow-up having 0.74 kg less weight gain, and those reporting more than 15 min/day cycling having ~1.5 kg less weight gain.  In women reporting cycling more than 15 min/day at baseline, who decreased their cycling, experienced greater weight gain (by ~2.1-2.5 kg, depending on extent of decrease) than those who maintained cycling at that level.  Women who reported cycling for more than 4 hours/week at follow-up had 29% lower risk of having gained more than 5% bodyweight over the follow-up period. |

**Supplementary Table 7. Summary of intervention studies evaluating the effects of changing cycling participation and health outcomes. Modified and updated from (58).**

| **Author, Date** | **Country** | **Study design** | **Participants** | **Intervention** | **Outcome measures** | **Key findings** |
| --- | --- | --- | --- | --- | --- | --- |
| Ostergaard (2012) | Denmark | Randomised controlled trial | 43 children, mean age 12 years, randomised into intervention (n=23) and control (n=20) groups. | Cycling to and from school for 8 weeks (84.2% compliance in intervention group) | Fitness (VO_2peak_), cardiometabolic risk profile score (based on fitness, body fat, blood pressure, blood lipids, insulin sensitivity) | Significant improvement in cardiometabolic risk profile in intervention compared with control group. No significant difference in change in VO_2peak_ between intervention and control groups, but significant association been total distance cycled and change in fitness. |
| Møller (2011) | Denmark | Randomised controlled trial | 48 adults, mean age 45 years, randomised into intervention (n=25) and control (n=23) groups. | Minimum of 20 minutes of daily commuter cycling for 8 weeks. | Fitness (VO_2max_), body fat, blood pressure | Significant increase in VO_2max_ and decrease in body fat in the intervention group compared with control group. No change in blood pressure. Relatively strong dose-response relationship (r^2^=0.46) between total amount of commuter cycling and change in VO_2max_. |
| De Geus (2009) | Belgium | Non-randomised intervention trial | 92 untrained healthy adults aged 30-65 years divided into intervention (n=74) and control (n=18) groups. 65 in the intervention group and 15 in the control group completed. | Cycling to work at least 3 times per week for 12 months, one-way distance 2-15 km | Fitness (maximal power and peak oxygen uptake (VO_2peak_) during a cycle ergometer test | Increases in maximal power and VO_2peak_ in the intervention group with a significant dose-response relationship between minutes of cycling per week and energy expended in cycling per week and change in VO_2peak_ |
| De Geus (2008) | Belgium | Non-randomised intervention trial | 92 untrained healthy adults aged 30-65 years divided into intervention (n=74) and control (n=18) groups. 65 in the intervention group and 15 in the control group completed. | Cycling to work at least 3 times per week for 12 months, one-way distance 2-15 km | Body mass, BMI, Fitness (maximum power and VO_2peak_), blood lipids, uric acid, C-reactive protein, blood pressure, quality of life (SF-36) | Significant increase in maximal power and VO_2peak_ in the intervention group compared with control group for group overall. No significant difference in body mass, BMI, blood variables or blood pressure between control and intervention groups. From the SF-36, vitality increased more in the intervention group than control group at 6 months, but this was not sustained to 12 months. |
| Hendriksen (2000) | Netherlands | Randomised controlled trial | 87 men and 35 women aged 25-56 years randomised into intervention (n=57) and control (n=58) groups. | Cycling to and from work (minimum one-way distance 3 km) at least 3 times per week for 6 months | Fitness (maximal power and maximal oxygen uptake (VO_2max_) during a cycle ergometer test) | Significant increase in maximal power and VO_2max_ in the intervention group compared with control group. |
| Oja (1991) | Finland | Randomised controlled trial | 38 men and 30 women aged 20-65 years randomised into intervention (n=26) and control (n=33) groups. | Cycling to and from work (average one-way distance 10 km) on average 3.75 times per week for 10 weeks | Fitness (VO_2max_ and time to exhaustion during an incremental treadmill test; blood lactate during submaximal exercise) | Significant increase in maximal power and VO_2max_, and decrease in blood lactate during submaximal exercise, in the intervention group compared with control group. |

**Supplementary Table 8. Summary of evidence on cycling and mental health, quality of life and wellbeing**

| **Author, Date** | **Location** | **Study design** | **Participants** | **Key outcome measures** | **Key findings** |
| --- | --- | --- | --- | --- | --- |
| Koolhaas (2018) | Rotterdam, Netherlands | Cross-sectional | 5554 adults, mean age 69 years | Health-related quality of life | Amongst those aged 65 years and older, high compared with low levels of cycling were associated with lower odds have having problems with mobility (by 37%), self-care (by 57%), daily activities (by 58%) and pain (by 33%). No relationship between cycling and aspects of health-related quality of life were observed amongst those aged under 65 years. |
| Ward (2018) | Southland, New Zealand | Cross-sectional | 775 adolescents aged 15-19 years | Life satisfaction | In females, but not males, cycling for transport was associated with higher life satisfaction |
| Aliva-Palencia (2017) | Barcelona, Spain | Cross-sectional | 788 adults who regularly travelled to work or study | Perceived stress | After adjustment for key confounders (age, sex, country of birth, number of employed people in household, chronic disease, self-perceived health, physical activity, public bicycle stations at work/study, bikeability at work/study), bikeability on commuter route), cycle commuting was associated 20% lower risk of reporting high perceived stress (on the Perceived Stress Scale (PSS-4)) compared with non-cycle commuting. |
| Brutus (2016) | Montreal, Canada | Cross-sectional | 123 adults employees from a single company | Stress and mood | Compared with commuting by car, cycling to work was associated with lower levels of stress during the first 45 mins of arrival at work, but was not associated with mood. |
| Mytton (2016) | Cambridge, UK | Prospective cohort | 801 adult commuters, median age 44 years | Physical wellbeing (PCS-8), mental wellbeing (MCS-8), sickness absence | Compared to not cycling to work, maintaining cycling to work for one year was associated with lower sickness absence (by ~ 1 day per year) in analyses adjusted for covariates and baseline levels of sickness absence.  Maintaining cycling to work for one year was also associated with higher physical and mental wellbeing, in unadjusted analyses. These associations were lost after adjustment for confounding variables, which included baseline levels of physical/mental wellbeing. |
| Rissel (2016) | Sydney, Australia | Cross-sectional | 473 adults who commuted to work | Satisfaction with transport, enjoyment of commute | Cyclists reported higher levels of enjoyment with their commute (52%) than car drivers (14%) or public transport users (10%). |
| Scheepers (2015) | Netherlands | Cross-sectional | 3075 adults | General health, psychological wellbeing | Those who chose to cycle, rather than use a car for short journeys (<7.5 km) had higher odds of perceiving that they were in general good health. No association between transport choice and perceived psychological wellbeing. |
| Crane (2014) | Sydney, Australia | Cross-sectional | 846 healthy adults aged 18-55 years | Quality of life (WHOQOL-BREF) | Cycling at least weekly (compared with cycling less than weekly) was associated with higher physical quality of life overall, and with higher levels psychological wellbeing in men. |
| Bopp (2013) | USA | Cross-sectional | 1234 employees aged over 18 years | Perceived health status | Cycle commuters had higher perceived health status than non-cycle commuters |
| Hendriksen (2010) | Netherlands | Cross-sectional | 1236 employees, median age 44 years, from large white-collar companies | Sickness absence | Compared to non-cyclists, commuter cyclists were absent for 1.3 fewer days per year (7.4 days vs 8.3 days). Compared with those who cycled shorter distances, those who cycled more often and for longer distances were absent for fewer days on average. |
| De Geus (2008) | Belgium | Non-randomised intervention trial | 92 untrained healthy adults aged 30-65 years divided into intervention (n=74) and control (n=18) groups. 65 in the intervention group and 15 in the control group completed. Intervention was cycling to work at least 3 times per week for 12 months, one-way distance 2-15 km | Quality of life (SF-36) | From the SF-36, vitality increased more in the intervention group than control group at 6 months, but this was not sustained to 12 months. |

**Supplementary Table 9** **Summary of peer-reviewed evidence on barriers to cycling in adults**

| **Author (Date)** | **Study design and population** | **Location** | **Cycling domains** | **Key findings** |
| --- | --- | --- | --- | --- |
| Fraser & Lock (2011) (118) | Systematic review, including 21 studies | USA -14 studies, Australia -3 studies, UK - 2 studies, Canada -1 study, Netherlands -1 study | Recreation  and transport | Systematic review of effects of the environment on cycling. Negative environmental factors were perceived and objective traffic danger, long trip distance, steep inclines and distance from cycle paths. Environmental factors positively associated with cycling included presence of dedicated cycle routes or paths, separation of cycling from other traffic, high population density, short trip distance, and proximity of a cycle path or green space. |
| Iwińska (2018) (119) | Cross-sectional survey of a random sample of 561 adults and a sample of 505 cyclists, plus nine in-depth interview and one focus group | Warsaw, Poland | Recreation and transport | The main perceived barriers were linked to lack of good cycling infrastructure, the feeling of insecurity due to the behaviour of drivers, bad weather and maintenance during winter. Cycling was mostly perceived as a leisure, rather than utilitarian, activity. |
| Manaugh (2017) (123) | Cross-sectional survey of 4944 university staff and students | Montreal, Canada | Transport | Presence of cycle paths was strongly associated with a higher frequency of cycling commutes. Safety, lack of secure cycle parking facilities, and high perceived effort were identified as barriers. |
| Macmillan & Woodcock (2017) (124) | Primary (stakeholder interviews and workshops) and secondary data used to develop a qualitative causal model of influences and outcomes of cycling | London, England  Nijmegen, Netherland  Auckland, New Zealand | Recreation and transport | The qualitative causal model demonstrated reinforcing loops of: increasing cycling participation leading to greater political will to improve the environment; increases in cycling numbers leading to improved safety (safety in numbers); and more cycling leading to normalisation of cycling in the population.  In Auckland and London (cities at or near start of the cycling growth trajectory), the real and perceived dangers of cycling were identified as the major limiting factor for growth in numbers, whereas in Nijmegen (with high cycling participation) cycling congestion and “market saturation” were considered important limiters to increasing cycling growth. |
| Swiers (2017) (120) | Cross-sectional survey of 194 university students aged 18-25 years | Liverpool, UK | Transport | Enjoyment and improving fitness were the main motivators for cycling, especially amongst regular cyclists, and saving money was also identified as a motivator. Bad weather and safety concerns were the main barriers, with lack of showers and cost of buying a bike also identified. Most felt there were more barriers to cycling than driving. |
| de Souza (2014) (121) | Cross-sectional survey of 288 college staff and students | Sao Paulo, Brazil | Transport | Perceived behavioural control (barriers) was assessed in relation to six possible obstacles to cycling: lack of infrastructure, lack of safety, distance, skill, hills and climate. The main deterrent to cycling was lack of adequate cycling infrastructure. Lack of safety (which was associated with inadequate infrastructure) and hills were other key barriers identified. |
| Engbers & Hendriksen (2010) (127) | Cross-sectional internet survey of 799 respondents aged 13-65 years | Netherlands | Transport | The main barriers for non-cyclists were perceived intensity of cycling (and being sweaty when arriving at work), living too far from work, bad weather and time constraints. Important facilitators were living close to work, health benefits and getting enough exercise. Relatively few non-cyclists identified environmental factors (e.g. paid parking, better/more cycle paths, better facilities at work) as facilitators. These data suggest that in countries with adequate cycling infrastructure (like the Netherlands) individual determinants are more important than environmental determinants of cycling behaviour. |
| Molina-Garcia (2010) (122) | Cross-sectional survey of 518 university students aged 18-25 years | Valencia, Spain | Transport | Both psychological and environmental variables were significant correlates of active commuting to University. There was a strong inverse relationship between access to private transport (car and motorbike) and active commuting. Presence and quality of walking and cycling facilities were positively related to active commuting. On an individual level, physical self-efficacy and perceived planning/psychosocial barriers were also significant correlates of active commuting. |
| de Geus (2008) (126) | Cross-sectional survey of 343 adults aged 18-65 years working outside the home, living within 10 km of work | Flanders, Belgium | Transport | Participants reporting social support from relatives who cycle, high levels of external self-efficacy and good cycle facilities at work were more likely to cycle for transport. A perception that cycling was cheaper and better for the environment was also associated with cycling behaviour. Lack of time (e.g. due to job and family commitments) and lack of interest were important barriers. Local traffic was not associated with participation in cycling to work. Thus, individual factors (social support, self-efficacy, perceived benefits and barriers) were more important than environmental factors as determinants of cycling in this context of good cycling infrastructure. |
| Titze (2008) (125) | Cross-sectional telephone survey of 1005 respondents aged 15-60 years. | Graz, Austria | Recreation and transport | Poor bike-lane connectivity and presence of steep elevations were negatively associated with cycling. Perceived barriers of physical discomfort, cycling being impractical (rain, clothes) were also negatively associated with cycling. Social support/modelling and perceived benefit of ‘rapidity’ were positively associated with cycling participation. |
